# Conserved intramolecular networks in GDAP1 are closely connected to CMT-linked mutations and protein stability

**DOI:** 10.1371/journal.pone.0284532

**Published:** 2023-04-14

**Authors:** Aleksi Sutinen, Dirk Paffenholz, Giang Thi Tuyet Nguyen, Salla Ruskamo, Andrew E. Torda, Petri Kursula

**Affiliations:** 1 Faculty of Biochemistry and Molecular Medicine & Biocenter Oulu, University of Oulu, Oulu, Finland; 2 Centre for Bioinformatics, University of Hamburg, Hamburg, Germany; 3 Department of Biomedicine, University of Bergen, Bergen, Norway; Consejo Superior de Investigaciones Cientificas, SPAIN

## Abstract

Charcot-Marie-Tooth disease (CMT) is the most common inherited peripheral polyneuropathy in humans, and its subtypes are linked to mutations in dozens of different genes, including the gene coding for ganglioside-induced differentiation-associated protein 1 (GDAP1). The main GDAP1-linked CMT subtypes are the demyelinating CMT4A and the axonal CMT2K. Over a hundred different missense CMT mutations in the *GDAP1* gene have been reported. However, despite implications for mitochondrial fission and fusion, cytoskeletal interactions, and response to reactive oxygen species, the etiology of GDAP1-linked CMT is poorly understood at the protein level. Based on earlier structural data, CMT-linked mutations could affect intramolecular interaction networks within the GDAP1 protein. We carried out structural and biophysical analyses on several CMT-linked GDAP1 protein variants and describe new crystal structures of the autosomal recessive R120Q and the autosomal dominant A247V and R282H GDAP1 variants. These mutations reside in the structurally central helices ⍺3, ⍺7, and ⍺8. In addition, solution properties of the CMT mutants R161H, H256R, R310Q, and R310W were analysed. All disease variant proteins retain close to normal structure and solution behaviour. All mutations, apart from those affecting Arg310 outside the folded GDAP1 core domain, decreased thermal stability. In addition, a bioinformatics analysis was carried out to shed light on the conservation and evolution of GDAP1, which is an outlier member of the GST superfamily. GDAP1-like proteins branched early from the larger group of GSTs. Phylogenetic calculations could not resolve the exact early chronology, but the evolution of GDAP1 is roughly as old as the splits of archaea from other kingdoms. Many known CMT mutation sites involve conserved residues or interact with them. A central role for the ⍺6-⍺7 loop, within a conserved interaction network, is identified for GDAP1 protein stability. To conclude, we have expanded the structural analysis on GDAP1, strengthening the hypothesis that alterations in conserved intramolecular interactions may alter GDAP1 stability and function, eventually leading to mitochondrial dysfunction, impaired protein-protein interactions, and neuronal degeneration.

## Introduction

The demand for sufficient energy supply *via* the aerobic process is elevated in neurons compared to other organelles and tissues, such as muscles [1]. Mitochondria are responsible for cellular respiration and linked to Ca^2+^ signalling and reactive oxygen species metabolism [2–5]. Since neurons depend on aerobic energy, their demand for oxidative phosphorylation is high, and 20% of the net oxygen consumed by the body is used for oxidative phosphorylation in neurons. Therefore, neurons are sensitive to alterations in mitochondrial function, and disruptions in mitochondrial dynamics can have severe consequences on neuronal functions.

Mitochondria are not isolated organelles, but interact with other cellular compartments, such as the endoplasmic reticulum, lysosomes, and peroxisomes, exchanging metabolites [2, 6, 7]. Mitochondria are renewed *via* fission and fusion, which are driven by proteins on the mitochondrial outer membrane (MOM), such as mitofusin 1 and 2 (MFN1/2), and the mitochondrial inner membrane (MIM), such as OPA1 and FIS1. Auxiliary proteins may bind to either MOM or MIM to enhance the process. The ganglioside-induced differentiation-associated protein 1 (GDAP1) is an integral MOM protein, proposed to have an auxiliary role in mitochondrial fission and fusion [8], possibly *via* redox-dependent interactions with cytoskeletal components [9]. However, the molecular basis of GDAP1 function and its exact relation to disease are currently not known.

Structurally, GDAP1 resembles glutathione *S*-transferases (GST), and it contains unique flexible loops [10, 11]. GDAP1 has two GST-like domains, followed by a transmembrane helix, which anchors the protein into the MOM. Structural data have shown a covalently bound dimer interface in GDAP1 [11, 12], and while dimerisation is a common feature in catalytic GSTs [13], the GDAP1 dimer is formed differently, with a unique interface having a central disulphide bridge between Cys88 from each subunit [11]. Enzymatic activity of GDAP1 has not been convincingly demonstrated, nor has any substrate been identified *in vivo*. Members of the GST superfamily share a common fold, but structural differences and low sequence conservation result in a diverse group of substrates; hence, it is possible that GDAP1 is an enzyme, but the substrate and reaction mechanism remain unidentified.

Increasing numbers of genes related to mitochondrial function have been linked to neuropathophysiological conditions. Inherited polyneuropathies are a genetically and clinically diverse group of neurodegenerative diseases, which affect the outer motor and sensory neurons in the peripheral nervous system (PNS) [14, 15], the most common being Charcot-Marie-Tooth disease (CMT). Clinical profiling divides CMT into three classes: demyelinating, axonal, and intermediate [16, 17]. The phenotype often implies insufficient mitochondrial fission and fusion, and mitochondria appear fragmented and elongated [6]. The etiology of CMT is linked to the hereditary pattern, whereby the autosomal recessive form has an earlier onset and more severe symptoms than the autosomal dominant form [18–20]. In the case of GDAP1, both autosomal dominant (axonal type CMT2) and recessive (demyelinating type CMT4) modes of inheritance are found, and disease severity is correlated with the location of the mutation in the protein. The *GDAP1* gene is one of the most common missense mutation targets linked to CMT [8, 21, 22]. GDAP1 is ubiquitously expressed in tissues, but most of the expression is confined to neuronal tissues [8, 23]. The most accurate structural data thus far cover the dimeric core GST-like domain of human GDAP1, including the GDAP1-specific insertion [11]. In addition, a structure of a construct missing the large GDAP1-specific insertion is available in monomeric form [10]. In full-length GDAP1, an amphipathic extension–originally termed the hydrophobic domain–links the transmembrane helix to the GST-like domain.

GSTs often contribute to mechanisms of neurodegenerative disease [24, 25]. GST superfamily members function in prokaryotic and eukaryotic metabolism by utilizing reduced glutathione to catalyse a range of chemically diverse reactions, and sequence conservation appears to be driven by fold stability instead of catalytic features, as reflected in the broad spectrum of GST substrates [26, 27]. Using X-ray crystallography and complementary biophysical and computational techniques, we carried out structural analysis on selected GDAP1 mutants linked to CMT. We also analysed GDAP1 sequence conservation to investigate its GST-linked ancestry and to get clues into its molecular function and the relationship between conserved residue interaction networks and disease mutations.

## Materials and methods

### Recombinant protein production and purification

The GDAP1Δ303–358 and GDAP1Δ319–358 constructs, with an N-terminal His_6_ tag and a Tobacco Etch Virus (TEV) protease digestion site, for producing soluble recombinant human GDAP1 in *E*. *coli*, have been described [11]. The point mutations R120Q, R161H, A247V, H256R, and R282H were generated in GDAP1Δ303–358, and the mutations R310Q and R310W in GDAP1Δ319–358, by a site-directed mutagenesis protocol with Pfu polymerase. All constructs were verified by DNA sequencing.

Recombinant GDAP1 variants were expressed in *E*. *coli* BL21(DE3) using autoinduction [28], and purified as described [11]. Briefly, GDAP1 was separated from the lysate by Ni^2+^-NTA chromatography, and the affinity tag was cleaved using TEV protease. Another Ni^2+^-NTA affinity step removed the tag and TEV protease. Size exclusion chromatography (SEC) was performed on a Superdex 75 10/300 GL increase column (Cytiva) using 25 mM HEPES (pH 7.5) and 300 mM NaCl (SEC buffer) as mobile phase. SEC peak fractions were analysed with SDS-PAGE and concentrated by centrifugal ultrafiltration.

### X-ray crystallography

Mutant GDAP1Δ303–358 crystals were obtained using sitting-drop vapour diffusion at +4°C. Proteins were mixed with mother liquor on crystallisation plates using a Mosquito LCP nano-dispenser (TTP Labtech). The protein concentration was 10–30 mg/ml in 75 nl, and 150 nl of reservoir solution were added. R120Q crystals were obtained in 0.1 M succinic acid, 15% (w/v) PEG 3350. A247V crystals were obtained in 0.15 M *DL*-malic acid (pH 7.3), 20% (w/v) PEG3500. R282H crystals were obtained in 0.1 M succinic acid, 20% (w/v) PEG 3350. For cryoprotection, crystals were briefly soaked in a mixture containing 10% PEG200, 10% PEG400, and 30% glycerol, before flash cooling in liquid N_2_.

X-ray diffraction data were collected at the PETRA III synchrotron (DESY, Hamburg, Germany) on the P11 beamline [29, 30] and the EMBL/DESY P13 beamline [31] at 100 K and processed using XDS [32]. The structure of wild-type GDAP1Δ303–358, PDB entry 7ALM [11], was used as the search model for molecular replacement in Phaser [33]. The models were refined using Phenix.Refine [34] and rebuilt using COOT [35]. The structures were validated with MolProbity [36]. The data processing and structure refinement statistics are in **Table 1**, and the refined coordinates and structure factors were deposited at the Protein Data Bank with entry codes 7B2G (R120Q), 8A4J (A247V), and 8A4K (R282H).

**Table 1:** Data processing and refinement statistics. Data in parentheses refer to the highest-resolution shell.

Variant	R120Q	A247V	R282H
Space group	P6_3_22	P2_1_2_1_2_1_	P2_1_2_1_2_1_
Unit cell	148.1, 148.1, 114.538 Å 90, 90, 120°	73.3, 115.7, 115.9 Å 90, 90, 90°	73.3, 113.4, 115.2 Å 90, 90, 90°
Resolution range (Å)	100–3.0 (3.1–3.0)	50–2.68 (2.84–2.68)	50–1.95 (2.07–1.95)
Completeness (%)	100 (100)	99.7 (99.3)	99.8 (99.9)
Redundancy	38.7 (39.0)	13.1 (13.5)	6.7 (6.7)
⟨I/σI⟩	17.0 (1.0)	16.9 (1.9)	15.4 (0.7)
R_sym_ (%)	27.3 (505.3)	8.8 (162.7)	4.9 (273.8)
R_meas_ (%)	27.7 (511.9)	9.1 (169.0)	5.3 (296.9)
cc_1/2_ (%)	99.9 (48.0)	99.8 (81.4)	99.9 (41.6)
R_cryst_/R_free_ (%)	23.0/26.5	25.2/28.3	22.5/25.1
RMSD bond lengths (Å)	0.002	0.015	0.014
RMSD bond angles (°)	0.5	1.5	1.4
MolProbity score / percentile	1.36 / 100^th^	2.04 / 97^th^	1.63 / 92^nd^
Ramachandran favoured/outliers (%)	95.1/0.00	96.4 / 0.2	97.9 / 0.4
PDB entry	7B2G	8A4J	8A4K

### Modelling

A model for full-length human GDAP1 was obtained from AlphaFold2 [37]. In addition, for an alternative model missing loops of the human wild-type GDAP1 crystal structure were built with CHARMM-GUI [38, 39]. Earlier structure-based bioinformatics results [12] were analysed further with respect to the mutational spectrum of GDAP1.

### Synchrotron small-angle X-ray scattering

The structure and oligomeric state of the GDAP1 mutants were analysed with SEC-coupled small-angle X-ray scattering (SAXS). SEC-SAXS experiments were performed on the SWING beamline [40] (SOLEIL synchrotron, Saint Aubin, France). Samples were dialyzed against SEC buffer and centrifuged at >20000 g for 10 min at +4°C to remove aggregates. 100 μl of each protein sample at 1.7–36 mg/ml were injected onto a BioSEC3-300 column (Agilent), run at a 0.3 ml/min flow rate. SAXS data were collected at +15°C, over a *q*-range of 0.003–0.5 Å^−1^. SAXS data analysis, processing, and modelling were done in ATSAS 3.0 [41]. Scattering curves were analysed and particle dimensions determined using PRIMUS [42] and GNOM [43]. Chain-like *ab initio* models were generated using GASBOR [44], dummy atom models were built with DAMMIN [45], and model fitting to data was analysed with CRYSOL [46].

To complement the SEC-SAXS, batch mode SAXS experiments were carried out for wild-type GDAP1 and four variants at the concentration range 1–4 mg/ml, to observe possible clear differences in oligomeric state at similar concentrations. These measurements were carried out on the P12 beamline at EMBL/DESY (Hamburg, Germany) [47].

### Synchrotron radiation circular dichroism spectroscopy

Synchrotron radiation circular dichroism (SRCD) spectra were collected from 0.5 mg/ml samples on the AU-SRCD beamline at the ASTRID2 synchrotron (ISA, Aarhus, Denmark). The samples were prepared in 10 mM HEPES pH 7.5 and 100 mM NaF buffer, equilibrated to room temperature in 0.1 mm closed circular quartz cuvettes (Suprasil, Hellma Analytics). SRCD spectra were recorded from 170 nm to 280 nm at +25°C. Three scans per measurement were repeated and averaged. The spectra were processed using CDToolX [48].

### Thermal stability

Thermal stability of GDAP1 variants was studied by nanoDSF using a Prometheus NT.48 instrument (NanoTemper), in SEC buffer. **NanoDSF** experiments were done at 0.5 mg/ml, and 3 aliquots of each sample were run simultaneously (technical replicates). Tryptophan fluorescence was excited at 280 nm, and emission was recorded at 330 and 350 nm, while the samples were heated from +20 to +90°C at a rate of 1°C/min. Changes in the fluorescence ratio (F_350_/F_330_) were used to determine apparent melting points. The sample analysis was done using Nanotemper PR.ThermControl analysis package utilizing Boltzmann fit analysis and average of three F_350_/F_330_ inflection points was taken. The curves presented in the figures are the averaged curves. The data were analyzed using Origin (OriginLab Corporation, Northampton, MA, USA).

### Sequence entropy

The NCBI non-redundant protein database was used for all sequence searches. Starting from the human GDAP1 reference sequence (NP_061845.2), iterative PSI-BLAST [49] searches were performed, initially accepting sequences with an *e*-value of 10^−99^ or less. In a second iteration, sequences with *e*-value < 10^-7^ were accepted, resulting in 5986 sequences. This initial set was divided into three subsets according to their annotations: (a) GDAP1, (b) GDAP1L1 and (c) GST-labelled. For many calculations, GDAP1 and GDAP1L1 were combined to improve the statistics of conservation calculations, as they form a clear subgroup within GST-like sequences. Since the separation was based on annotations, sequences labelled as hypothetical or putative were removed. Sequence calculations were done either on subgroups or across the full set of 5065 sequences.

All multiple sequence alignments were calculated with MAFFT [50]. Results are always considered with respect to a reference sequence. This was done by removing any columns corresponding to a gap in the reference. This does lose information but is effectively essential for interpretation. For the GDAP1L1 group, the reference was the human sequence. For the larger GST group, the sequence of PDB entry 1PKZ was used to allow interpretation in structural terms.

Per-site entropy at site *i* in a multiple sequence alignment was given by

$$S_{i}=\Sigma_{j=1}^{20}\;\log_{20}\;p_{j}$$

where *p_j_* is the probability of seeing amino acid type *j* at the site and the summation runs over the 20 amino acid types. Gaps were treated as missing data. The use of base 20 in the logarithm ensures that *S* ranges from 0 (fully conserved) to 1 (random). In the interpretation below, *S*<0.2 was considered conserved and *S*<0.1 as highly conserved.

### Kullback-Leibler divergence

The most interesting sequence sites are those which are conserved within groups of proteins, but different between groups. The Kullbach-Leibler divergence (*D^KL^*) captures the difference between two distributions. With discrete distributions (types of amino acids), the value at position *i* is given by

$$D_{i}^{KL}=\Sigma_{j}^{20}p_{i,j}\;\ln\frac{p_{i,j}}{q_{i,j}}$$

where the summation runs over the 20 amino acid types. *p_i,j_* and *q_i,j_* are the frequencies (probabilities) of seeing residue type *j* at position *i* in the first and second protein groups [51, 52].

### Phylogenetic analyses

Maximum likelihood phylogenetic trees were calculated with MrBayes [53, 54] with four independent chains of length 2 × 10^6^ steps, with 25% of the steps discarded for burn-in and fixed amino acid exchange rate matrices. The trees in results are all consensus/average trees displayed with Interactive Tree of Life (iTOL) [55] using a bacterial sequence (WP_173192278.1) to root the tree.

Sequence data sets for phylogeny were different to those used for conservation calculations, the aim being to cover sequences from GDAP1 to common GSTs and archaea. The first sequence searches and guide trees had identified the archaeal and bacterial sequences closest to GDAP1. The similarity matrix suggested that bacterial GSTs were remotely related to both the eukaryotic and archaeal sequences. The first 799 database hits of GDAP1 formed one group, and the first 1000 hits from human GDAP1L1 (NP_001243666.1) the next. The first 1000 hits from the bacterial GST (TNE50161.1) and the human GST (sequence from PDB entry 1PKZ) were the third group. Finally, the first 1000 hits for the archaeal sequence (MAE98075.1) formed a fourth group.

Each of these four sets were then reduced to 100 representatives based on the similarity matrix calculated during a sequence alignment [56]. The procedure sorts the distance matrix to get the closest sequence pairs and removes one member at a time, ensuring the most even possible spread of sequences. Combining the 4 × 100 sets and removing duplicates left 397 sequences used for phylogeny.

## Results

**Fig 1:**
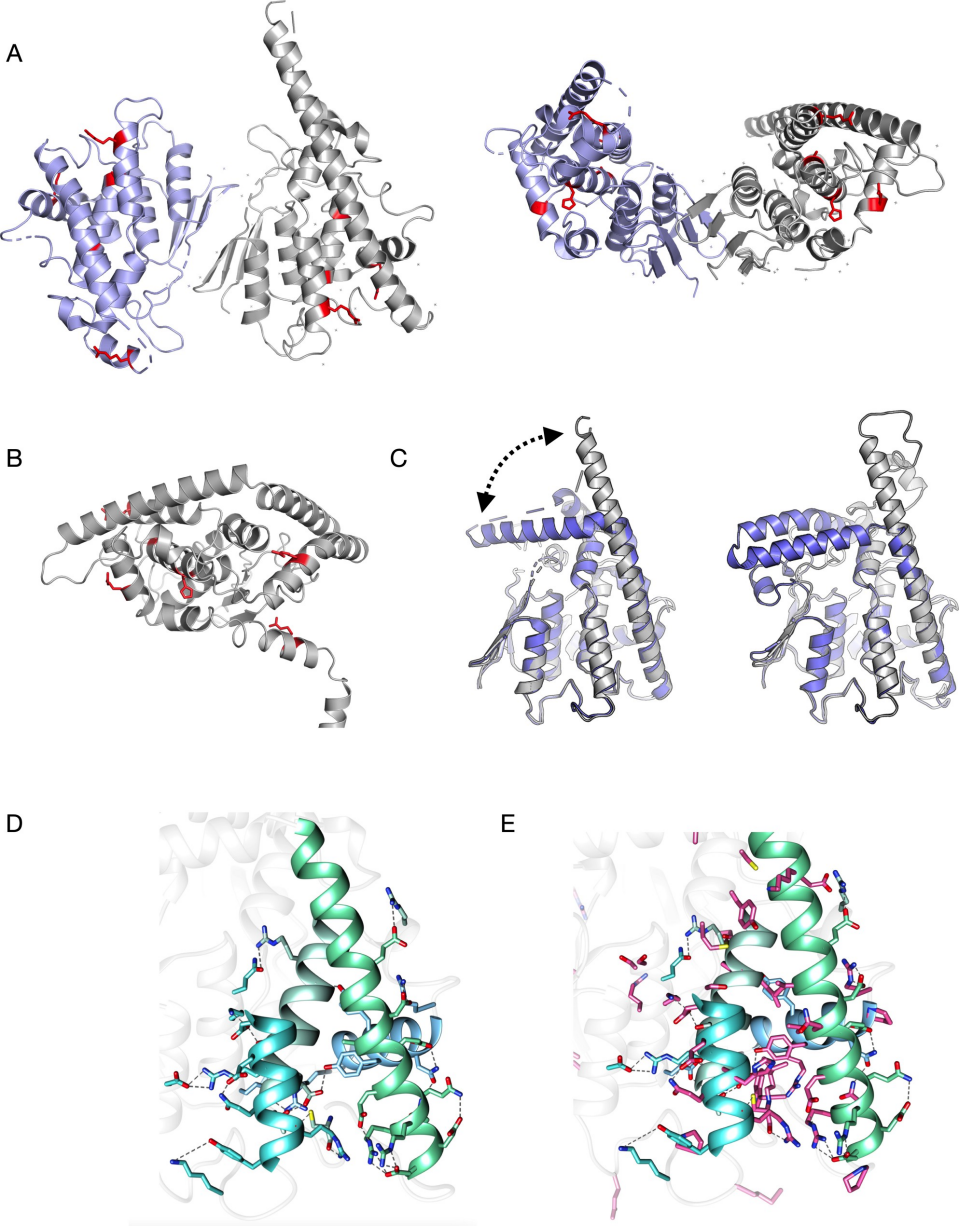
Overall structure of GDAP1. A. Location of the mutations studied here, mapped onto the crystal structure of wild-type GDAP1 [11] in two different orientations. At the centre of the dimer interface, a disulphide bridge between Cys88 from each protomer links the dimer covalently. B. The mapping of the mutations studied here (red) onto the AlphaFold2 model, to include those not visible in the crystallised construct (R161H, R310Q, R310W). The TM domain is at the bottom right, and the helix preceding it harbours Arg310. C. Open/close conformations involving the long helix ⍺6 have been observed both experimentally (left) and using structure prediction (right). Closed conformations are in blue and open in grey. D. Hydrogen bonding network of residues on the core helices of GDAP1. E. Same view as D, but known sites of CMT mutations have been added in magenta. CMT mutations are clustered on the GDAP1 core helices.

Building upon earlier work on GDAP1 structure [10–12], we focused here on several CMT-linked variants that reside on different secondary structure elements in the GDAP1 3D structure. While we earlier specifically looked at R120W and H123R on helix ⍺3 [12], here we characterised the variants R120Q, R161H, A247V, H256R, R282H, R310Q, and R310W. The stability and solution structure were studied for all variants, while crystal structures were determined for three of them: R120Q, A247V, and R282H. The location of the mutation sites in the GDAP1 structure is shown in **Fig 1A and 1B**.

### Helices α3, α6, α7, and α8 form a scaffold for GDAP1 intramolecular networks

The majority of CMT-linked missense mutations in GDAP1 are located within the vicinity of the hydrophobic clusters of the GST-like domains and the dimer interface [11], and the variants may induce changes in intramolecular hydrogen bonding networks [12]. In addition to the R120W and H123R studied earlier [12], we determined three new mutant crystal structures: R120Q, A247V, and R282H. These mutations reside in helices α3 (R120Q), α7 (A247V), and α8 (R282H), which are core elements of the GDAP1 fold. We shall first look at the central helices regarding GDAP1 folding.

The GST-like core fold of GDAP1 is supported by the α7 helix, surrounded by helices α3, α6, and α8. The helix α3 is connected to α6 *via* the α6-α7 loop, and Cys240 in this loop–itself being a CMT mutation site–is central to many interactions. The α6 helix can either extend or turn back towards the dimer interface, as seen in earlier crystal structures and models [12]. A new model built here indicates that the extended conformation is predictable (**Fig 1C**). Open/close movements of α6 can be functionally relevant for GDAP1 interactions with other proteins, such as cofilin and tubulin [9]. The α8 helix is positioned perpendicular to the others, and its orientation could be related to the transmembrane helix position, as it is expected to face the mitochondrial outer membrane surface. Together, these helices form an intramolecular network of polar (**Fig 1D**) and non-polar contacts, and many CMT-linked missense mutations are found on these helices (**Fig 1E**).

There are 15 designated hydrogen bond contacts or ionic interactions between helices α3, α6, α7, and α8. Helix α7, which is central to the GDAP1 fold, makes only a few hydrogen bonds to the surrounding helices, and hydrophobic residue clustering houses the α7 helix in the core of the fold. This is in line with earlier centrality analyses of the GDAP1 fold, showing helix α7 to be the most central part of the 3D structure [12]. Some of the disease mutations on helices α3, α6, and α8 correspond to solvent-exposed residues, and these helices show more polar interactions and higher flexibility compared to α7.

### Structural effects of the individual CMT mutations

**Fig 2:**
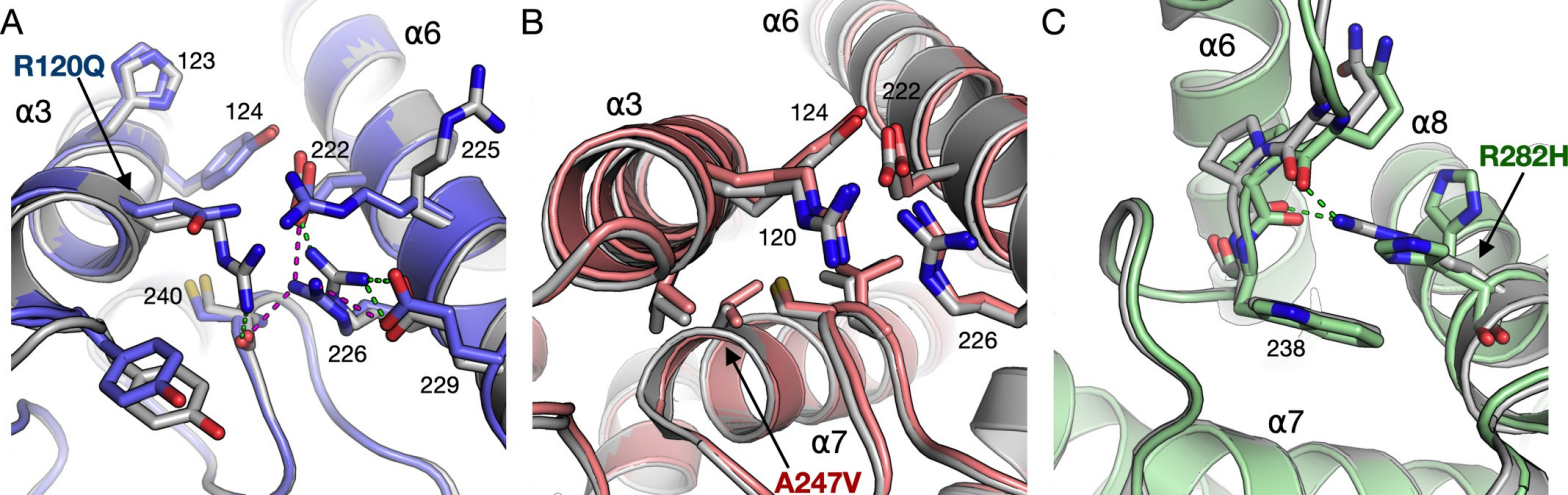
Structural details for each point mutation studied by X-ray crystallography in this study. A. Immediate environment of the R120Q mutation. Arg120 participates in a hydrogen bond network between helices α3 and α6. B. Effects of the A247V mutation. Ala247 on helix α7 is a central residue of the GDAP1 hydrophobic core. C. The R282H mutation. Arg282 interacts with the α6-α7 loop, and the mutation causes loss of these interactions. The structure of wild-type GDAP1 is shown in grey in all panels.

We have previously analysed the effects of R120W and H123R at the protein level, both residing on helix α3 [12]. Here, we extend the human GDAP1 crystal structure analyses to three more CMT mutations: R120Q, A247V, and R282H (**Fig 2, Table 1**).

The R120Q mutation is located close to the N-terminal end of the α3 helix. Due to the mutation, residue 120 loses contact with the α6-α7 loop tip at Cys240, whereby the hydrogen bond between the Arg120 side chain and the backbone carbonyl of Cys240 is lost (**Fig 2A**). This is further linked to alterations in surrounding side chain conformations.

The A247V mutation site resides near the N terminus of helix α7, tightly surrounded by helix α3 and the α6-α7 loop, and having van der Waals contacts to Val121, Tyr124, Cys240, and Thr245. Comparing to the wild-type protein, changes in the crystal structure are small, but an overall movement of surrounding protein segments is caused by the presence of Val in this position, due to the increased volume of the side chain. Both helices α3 and α6 move slightly away, without altering hydrogen-bonding patterns (**Fig 2B**).

R282H is located on helix α8, and the side chain of Arg282 points inwards in the wild-type GDAP1 structure; it is stacked against Trp238 and makes hydrogen bonds to the backbone carbonyl groups of residues 236 and 237 in the α6-α7 loop (**Fig 2C**). All these interactions are lost upon the mutation, and the His residue in the mutant is observed in a double conformation, making no side-chain hydrogen bond contacts.

### Conformation and stability in solution

Earlier computational predictions suggested an overall destabilising effect of CMT-linked mutations in the GDAP1 protein [12]. Similarly, protein destabilisation was observed experimentally for the myelin protein P2 in the context of all identified CMT mutations [57, 58]. A comparative analysis of seven GDAP1 variants in solution was therefore carried out here, to support the crystal structures and other complementary data from the current and earlier studies, and to identify general trends linking CMT mutations and GDAP1 stability *in vitro*. While the 3D shape and dimensions were studied using SAXS, SRCD was employed to follow secondary structure content and nanoDSF to compare thermal stability.

**Fig 3:**
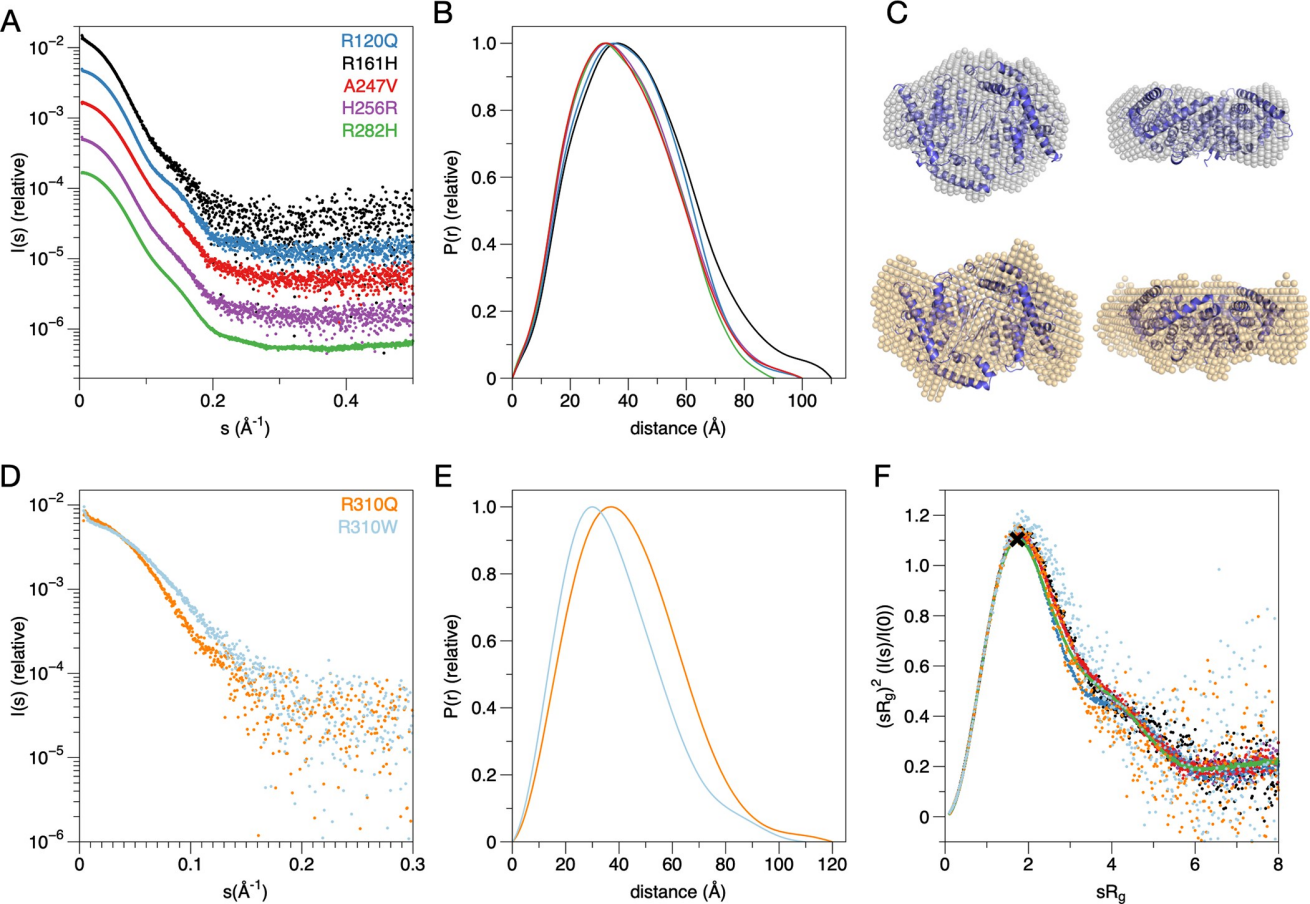
SAXS analysis. A. SAXS curves for the mutants in the GDAP1Δ303–358 construct. The curves have been displaced along the y axis for clarity. B. Distance distributions for the curves in A. R161H shows a more open conformation than the other variants. C. Top: Dummy atom model of R282H superimposed with a structure based on the collapsed conformation of wild-type GDAP1Δ295–358 [12]. Bottom: Dummy atom model of the R310Q mutant in GDAP1Δ319–358, superimposed on the same structure, indicating additional volume for the C-terminal extension. D. SAXS curves for the mutants in the GDAP1Δ319–358 construct. The dominant monomer peak from SEC was used for R310W and the dimer peak for R310Q. E. Distance distributions for the curves in D. F. Dimensionless Kratky plots for all constructs show similar levels of rigidity and globularity. The cross marks the location of the peak in a perfect globular particle.

The SAXS analysis of GDAP1Δ303–358 showed that the protein particle dimensions in solution correspond to a dimer, and the scattering profiles showed only minor shape differences, ruling out large-scale conformational differences or aggregation (**Fig 3A, Table 2**). All variants in this construct background eluted mainly as dimers, and the top of the peak was selected for the analyses. The largest deviation was observed for R161H, which had a larger R_g_ than the other variants, suggesting a more open structure (**Fig 3B**). Also R120Q had a slightly different conformation, most clearly seen in the distance distribution. The pair distance distribution function showed that the maximum particle dimension in all samples was ~90–110 Å, which corresponds to a dimer (**Fig 3B**). *Ab initio* modelling, here carried out on the R282H mutant, which was measured at the highest concentration, supported the presence of a dimeric GDAP1, corresponding to the conformation of the dimer we previously showed to fit the solution SAXS data for wild-type GDAP1 [12] (**Fig 3C**). As the SEC-SAXS experiments were carried out at the maximum concentration for each variant, we complemented these experiments with a set of batch-mode SAXS experiments with wild-type GDAP1 and four mutant variants (**S1 Fig**). All variants in this supplementary experiment are predominantly dimeric in the concentration range 1–4 mg/ml.

**Table 2:** SAXS parameters. Data for wild-type GDAP1 as well as the monomeric mutation Y29EC88E are taken from [11]. The MW estimate corresponds to the Bayesian estimate from PRIMUS.

Variant	Δ303–358 dimer/ Y29EC88E monomer	R120Q	R161H	A247V	H256R	R282H	R310Q main peak	R310W main peak	Δ319–358 dimer/monomer
R_g_ (Guinier) (Å)	30.7/24.5	30.9	33.4	29.8	30.3	29.3	33.3	29.3	34.7/27.1
R_g_ (GNOM) (Å)	30.6/24.59	30.7	33.6	30.0	30.3	29.4	33.6	29.9	33.5/27.3
D_max_ (Å)	99/86.7	99.7	110	100	99.2	91.0	120	110	107.9/89.6
V_p_ (nm^3^)	105.8/58.7	102.0	127.4	93.1	95.8	89.4	101.2	58.2	129.5/69.4
Estimated MW (kDa)	72.4/35.4	67.1	91.2	62.4	62.4	59.5	94.2	58.2	94.2/46.7

For the two mutations affecting Arg310, we used the longer construct GDAP1Δ319–358, and both R310Q and R310W eluted as two peaks in SEC-SAXS, corresponding to a dimer and monomer (**Fig 3D, 3E and Table 2**). This behaviour has previously been linked to protein concentration, in that GDAP1 monomers can be observed at low concentration [11], and the Arg310 mutants were here studied at lower concentration than all the other mutants. R310Q mainly eluted as a dimer, while the highest peak for R310W was monomer; since the protein concentrations were similar, this result indicates that R310W may affect GDAP1 dimerisation and that the dimerisation equilibrium is rather slow. The SAXS data for the dominant peak for each mutant were analysed further. The R310Q dimer was more elongated than R282H of the shorter construct (**Fig 3C**), reflecting the presence of the C-terminal amphipathic domain in the construct. Comparing the Kratky plot for all variants studied here, it is evident that they all are rigidly folded, with little flexibility (**Fig 3F**).

**Fig 4:**
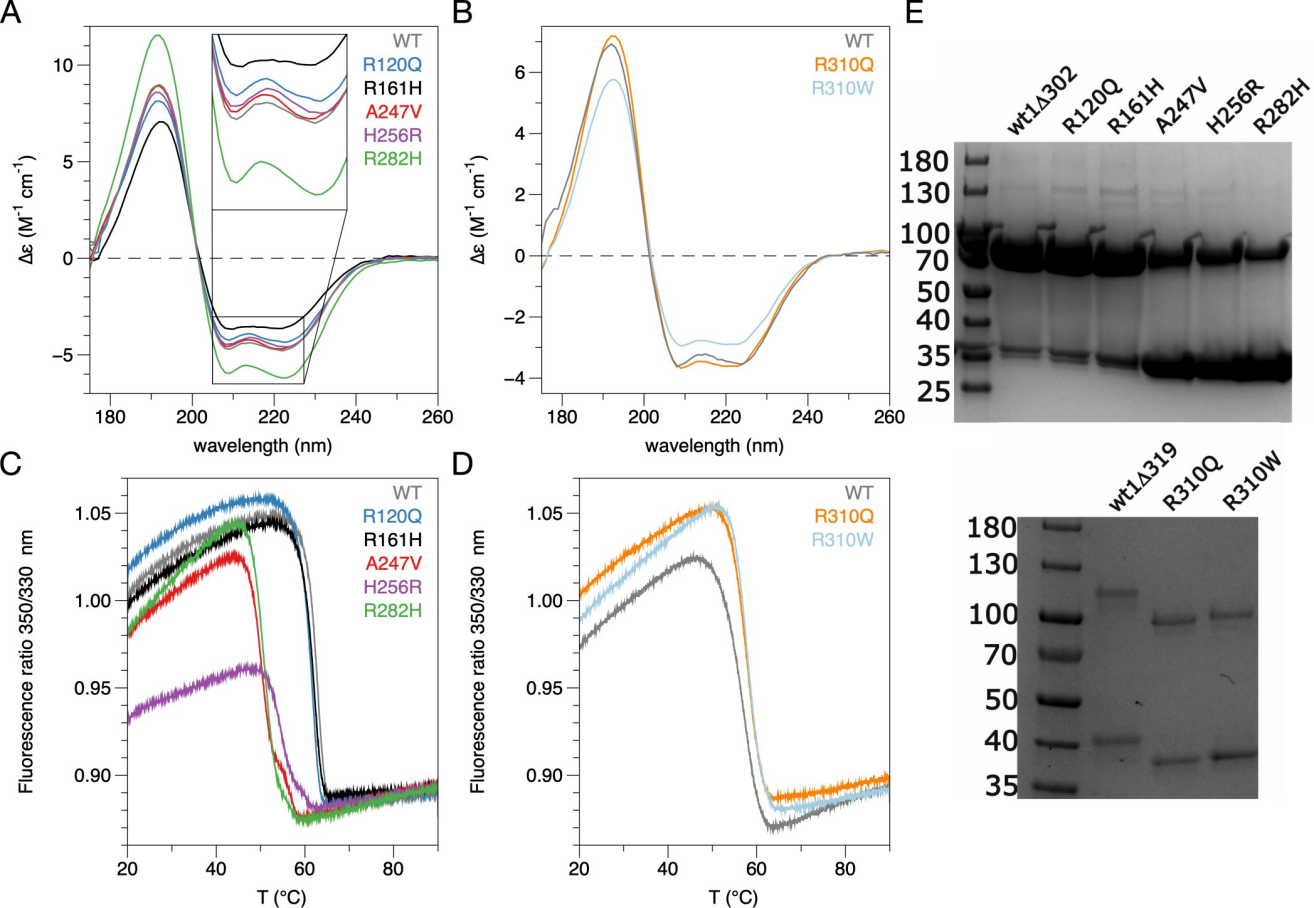
Folding and stability of GDAP1 as affected by CMT mutations. A. SRCD spectra for the mutants in the GDAP1Δ303–358 construct. The spectral shape is most different for R161H, and for R282H, the spectral amplitude is increased, but the shape does not change compared to wild-type GDAP1. B. SRCD spectra for the mutants in GDAP1Δ319–358. C-D. nanoDSF analysis for the mutants in GDAP1Δ303–358 (C) and GDAP1Δ319–358 (D). The curves shown are each average of three independent nanoDSF curves run in parallel. E. Non-reducing SDS-PAGE analysis of all studied variants. Top: variants in GDAP1Δ303–358; wt1Δ302 refers to the wild-type construct. Bottom: variants in GDAP1Δ319–358; wt1Δ319 refers to the wild-type construct. For the longer constructs (panel below), the added segment is most likely a membrane-binding motif, and the mutations may affect membrane interactions; this could explain the difference in electrophoretic mobility between the wild-type and mutant variants. The segment in question is likely to bind to SDS and form a helical structure instead of getting denatured. The uncropped gel images are in **S2 Fig**.

For thermal stability and secondary structure analysis, nanoDSF and SRCD were performed. The SRCD spectra showed similar secondary structure composition for all variants and wild-type GDAP1, indicated by the fact that the spectral shapes were nearly identical, despite some fluctuation in amplitude. The latter could be caused by errors in concentration determination or aggregation status in the strong synchrotron UV beam, or by differences in dynamics of the secondary structure elements. The SRCD spectrum for R161H showed a slight change in the spectral shape between 205–225 nm, which could be linked to its more open conformation observed in SAXS above. It can be concluded that none of the studied mutations interfere strongly with the overall folding of GDAP1 (**Fig 4A and 4B**). The nanoDSF experiment (**Table 3**) revealed a ~1–12°C decrease in apparent melting temperature for all core domain mutants, compared to the wild-type GDAP1Δ303–358 (**Fig 4C**). This is similar to the previously studied R120W and H123R mutants, which had melting temperatures ~52–54°C, *i*.*e*. a drop of ~10°C compared to the wild-type protein [12]. Notably, the F_350_/F_330_ ratio varies between the variants at the starting point, especially so for H256R, which may be an indication of subtle differences in structure. All in all, the biophysical data indicate that the structural effects of all studied CMT variants in the core domain are local, and the mutations do not cause large-scale conformational changes at a level detectable with SRCD or SAXS. However, all studied mutations in the core domain caused a decrease in thermal stability, suggesting a breakdown of stabilising intramolecular interactions within the GDAP1 molecule. As the most dramatic example, the mutation A247V inside the folded core decreased the stability by 13°C, indicating the importance of a correctly packed hydrophobic core for GDAP1 stability.

**Table 3:** nanoDSF apparent melting points. All values are average ± standard deviation from 3 replicates.

Sample	T_m_ (°C)
GDAP1Δ303–358	62.6 ± 0.01
R120Q	61.3 ± 0.03
R161H	61.8 ± 0.05
A247V	49.6 ± 0.02
H256R	54.6 ± 0.07
R282H	50.8 ± 0.02
GDAP1Δ319–358	56.9 ± 0.03
R310Q	57.9 ± 0.02
R310W	57.8 ± 0.06

For the mutations at position 310, it was observed that both R310Q and R310W in fact increased the stability of GDAP1Δ319–358 by ~1°C (**Fig 4D**). This can be explained by the fact that the segment carrying these mutations is no longer part of the core domain, but rather likely to represent an ⍺ helix attached to the membrane surface, possibly linking the membrane to the core domain. Overall, GDAP1 stability correlates with the dimer-monomer ratio observed in solution; the most destabilised mutants also show a higher fraction of monomeric protein on non-reducing SDS-PAGE (**Fig 4E**), even though in the 3D structure, they are far from the dimer interface. The exact mechanism for this observation is currently unknown, but it could mean that altered protein dynamics and local conformations affect the dimer formation and/or that dimerisation in turn stabilises the protein fold.

### Implications of the point mutations in the context of the whole GDAP1 protein

**Fig 5:**
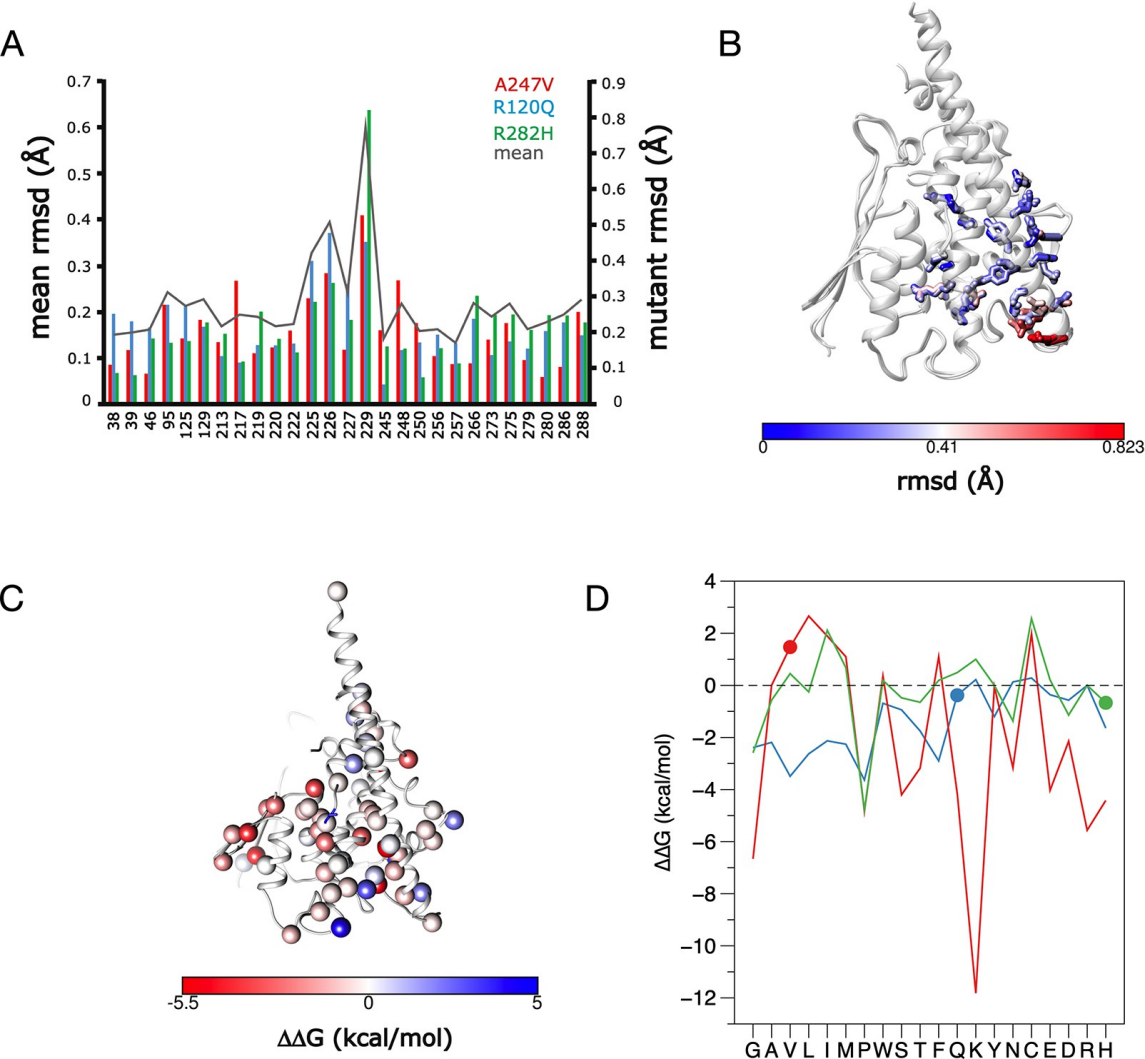
Structural bioinformatics analysis of the crystallised CMT variants. A. The C⍺ deviation of each mutant vs. wild-type GDAP1 structure. The highest deviations can be found in the residue range 220–230. Shown are only the residues participating in the hydrogen bonding network. B. Mapping the results onto the structure, it becomes evident that the C-terminal end of helix ⍺6 deviates the most from wild-type GDAP1 on average. C. Average predicted ΔΔG effect of GDAP1 mutations at CMT sites, as defined by CUPSAT. D. CUPSAT predictions for R120Q, A247V, and R282H being mutated into all possible amino acids. Note that a negative ΔΔG in CUPSAT means destabilisation, and that A247V is falsely predicted as stabilising. The mutations studied here are marked with spheres. Ala247, red; Arg120, blue; Arg282, green.

The studied mutations cause subtle variations in hydrogen bond and van der Waals distances in nearby residues in the crystal structures, while there are no drastic structural differences, when the mutant structures are superposed onto the wild-type crystal structure. However, when comparing the variation of the residues in helices α3, α6, α7, and α8, in comparison to the wild-type Cα-atoms, a specific pattern arises. We analysed the residues participating a hydrogen-binding network in the mutant structures (**Fig 5A**), and the mapping shows that the residues pointing towards the dimer interface have only minor structural variations compared to the wild-type protein. In contrast, at the C-terminal end of helix α6, in all mutant crystal structures, the variation of the Cα atoms is high (**Fig 5B**). This suggests that flexibility of helix α6 could arise from altered intramolecular contacts in the vicinity. The C-terminal end of the α6 helix, the conformation of which apparently is affected by the mutations studied here, is itself a target for multiple CMT mutations, affecting Gln218, Va219, Glu222, Arg226, and Glu227 [18, 59–62].

We additionally investigated the more global mutational effects using bioinformatics tools. In our previous study [12], we analysed the wild-type GDAP1 core domain structure and the full-length AlphaFold2 coordinates with CUPSAT and MAESTRO [63, 64]. This provided predicted ΔΔG values and geometric properties; the results from CUPSAT analyses are further depicted in **Fig 5C**. On the average, most CMT mutation sites are predicted to cause destabilisation and in general, the few mutations predicted to be stabilising lie on the protein surface.

While the above analyses indicate an average effect that may destabilise GDAP1 structure, we looked at predictions for each mutation crystallised here in more detail (**Fig 5D**). In essence, all substitutions to Arg120 are unfavourable; this shows that the interactions made by Arg120 are important for folding and stability. This is in line with our experimental data for both R120Q and R120W, which indicate minor changes in structure, but destabilisation of the fold. For Ala247, a mutation into Val is predicted to be slightly stabilising, indicating that such a replacement, in a tightly confined pocket and with potential long-range effects, is difficult for the prediction algorithm. In the case of Arg282, mutation to His is indeed one of the most destabilising variants in the prediction, linked to a loss of several key interactions by the Arg282 side chain.

### Phylogenetic analysis of GDAP1 and the GST superfamily

The ubiquitous GST superfamily is inherently very large and diverse. There are massive differences between families and even within the subfamilies. Starting a BLAST search with the reference sequence will quickly end up in GSTs. This means that GSTs will swamp other proteins in any naïve analysis. To avoid this, GDAP1, GDAP1L1 and GSTs were considered separately.

**Fig 6:**
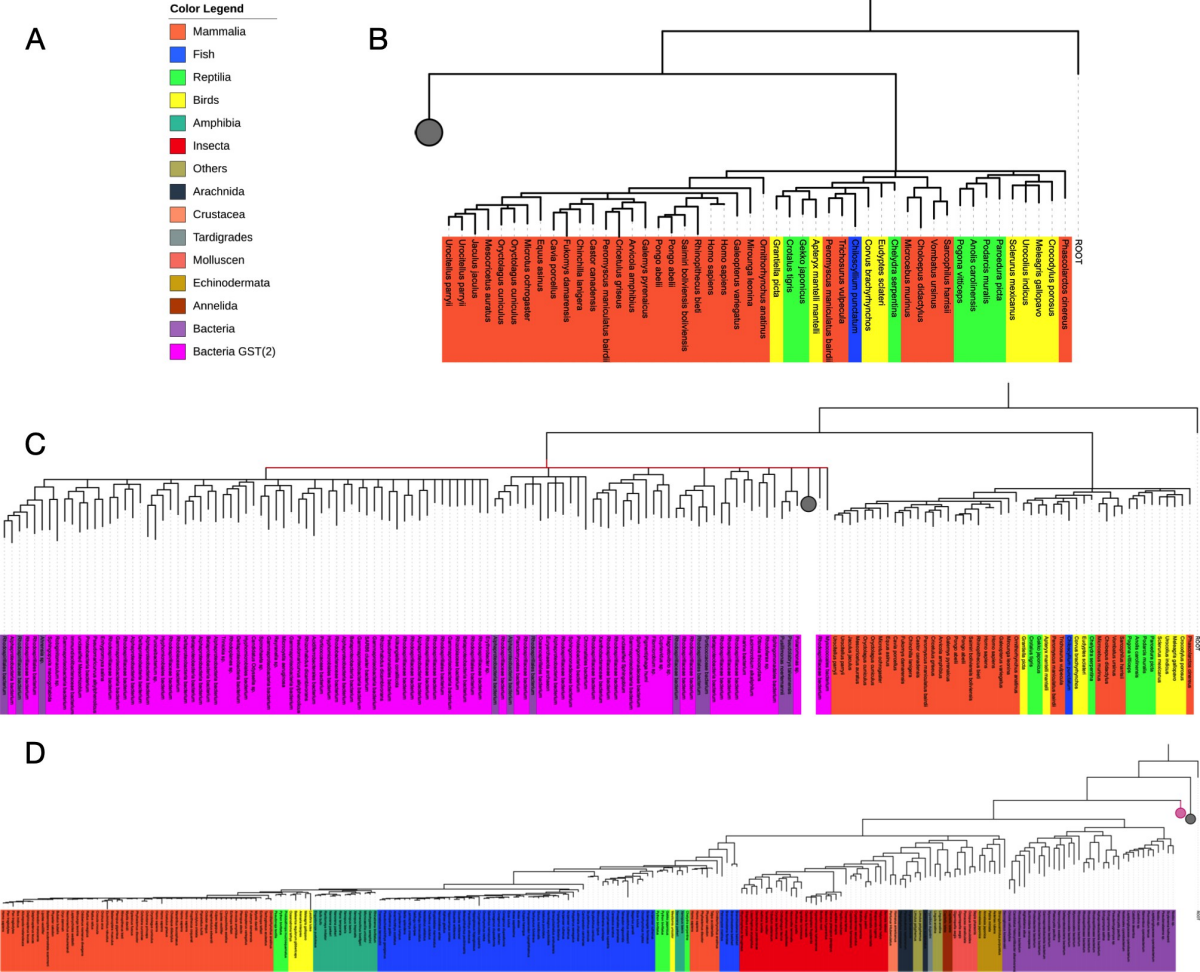
Phylogenetic analyses. A. Colour scheme for phylogenetic trees. Note that not all organisms seen in the colour legend are present in every picture. This is because the trees are very large and to increase visibility parts of the tree will be collapsed (denoted by a grey circle), to hide underlying branches. B. The first branch of the phylogenetic tree. The tree was rooted at the root sequence. On the right side are eukaryotic GST sequences (mammals, fish, birds, reptiles). The branch includes 48 sequences. The rest of the 398 sequences that are represented are at hierarchically lower levels of the phylogenetic tree. The corresponding branches were collapsed (grey circle). C. In contrast to panel B, the left branch is partially extended. The right-sided branch shows eukaryotic GST sequences, while the left side shows a total of 110 bacterial GST sequences. The remaining sequences are at hierarchically lower levels of the phylogenetic tree. The corresponding branches were collapsed (grey circle). The red line indicates that the time could not be resolved at this level. D. The last hierarchical level of the phylogenetic tree. The tree was rooted at the root sequence. Purple sequences on the right side correspond to bacterial GST sequences. Sequence for eukaryotic GDAP1 and GDAP1L1 are in the left side branches. The grey circle denotes the collapsed branch for the eukaryotic GSTs (see panel B) and the magenta circle denotes the second group of bacterial GST sequences (see panel C).

The first calculations focussed on GDAP1 (**Fig 6**). Intriguingly, based on large-scale sequence alignments, GDAP1 turns out to be more closely related to prokaryotic GSTs than eukaryotic proteins. In this situation, we must remember that sequence conservation across the GST superfamily is overall low. The phylogenetic calculations do not resolve to a clear bifurcating consensus tree, and the uncertainty is very high around the divergence of the GDAP1 group. One can say that GDAP1 is at least as far from generic eukaryotic GSTs as it is from prokaryotic proteins. The analysis provides little clues as to any enzymatic function of GDAP1, but it supports the findings that it has no conventional GST activity.

**Fig 6B and 6C** show that eukaryotic GSTs split off from other GSTs very early in evolutionary history. This was not to be expected. In addition, **Fig 6C** shows that the evolutionary history at this level could not be resolved (represented by a red line) despite sampling over millions of MCMC steps. **Fig 6D** shows the last level of the phylogeny, where the consensus does converge to a proper bifurcating tree. Towards the right side, there is a branch of bacterial GST sequences (purple), and in the left branch, the GDAP1/GDAP1L1 sequences are found again. The figure also shows that orthologs of GDAP1 and GDAP1L1 are found in mammals, birds, amphibians, and fishes and likely orthologs of those genes in invertebrates as well.

One may well want to interpret the results in terms of the endosymbiotic theory and remember that archaea are closer to eukaryotes than bacteria [65]. Since GDAP1 is an outer mitochondrial membrane protein, one might expect it to be more closely related to archaeal proteins. The tree, however, does not confirm or refute this. Calculating for longer simply generates more tree topologies with similar probabilities. Ultimately, one might admit that the history of the protein family involves too many duplication or gene transfer events to fit neatly into a simple timeline.

### Sequence entropy in GDAP1

**Fig 7:**
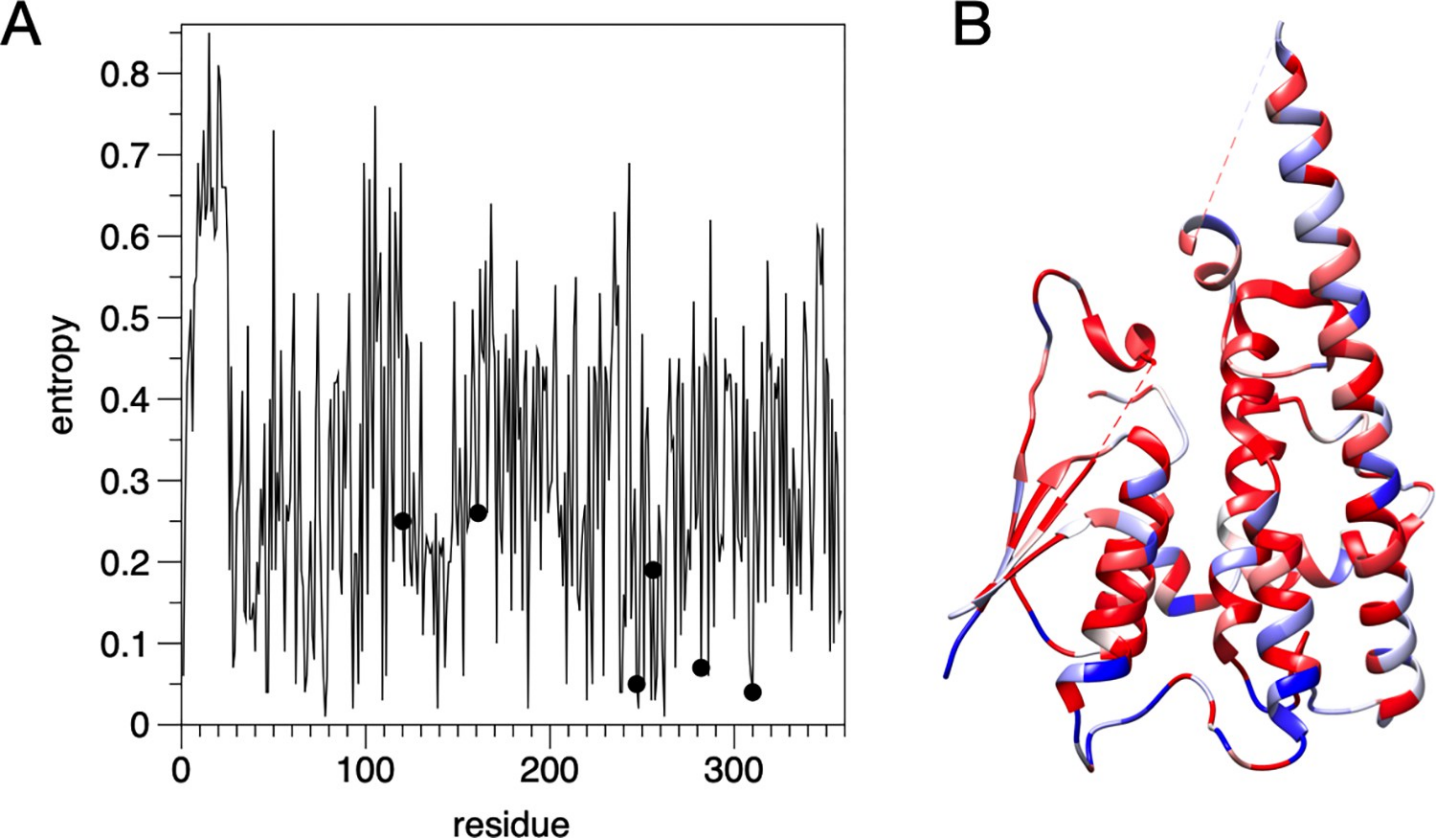
Sequence entropy analysis. A. Entropy plot for GDAP1. The positions of the mutations studied here are shown with dots, and these positions indicate a high level of conservation (low entropy). B. Mapping of entropy onto the GDAP1 crystal structure monomer. Blue indicates high entropy and red low.

Sequence conservation (entropy) calculations were carried out on the different groups to identify their conserved residues and sites which differ between the groups. We first consider the GDAP1 subset of sequences (**Fig 7A**). 44 residues were found with an entropy score ≤ 0.10 (highly conserved) and a fraction of non-gap characters in the alignment > 70% (**Table 4**); the entropy is mapped onto the GDAP1 structure in **Fig 7B**. The data highlight several interesting residues, which are known to be sites of CMT mutations, relevant for folding and structure, or with possible functions in ligand binding. These aspects are discussed more below.

**Table 4:** Entropy analysis of the GDAP1 subfamily. Shown are the positions (human GDAP1 reference sequence numbering) with the lowest entropy (S < 0.1).

residue	entropy	frac	Amino acid	residue	entropy	frac	amino acid	residue	entropy	frac	amino acid
28	0.07	0.78	L	98	0.09	0.94	E	248	0.02	0.97	D
29	0.09	0.78	Y	109	0.03	0.87	L	251	0.05	0.97	L
40	0.09	0.83	V	111	0.06	0.91	P	255	0.03	0.97	L
46	0.04	0.83	E	139	0.02	0.91	G	257	0.03	0.97	R
47	0.04	0.83	K	143	0.07	0.91	H	258	0.06	0.97	L
56	0.09	0.83	V	153	0.06	0.91	P	261	0.09	0.97	L
62	0.05	0.84	E	171	0.1	0.94	L	262	0.01	0.96	G
67	0.04	0.84	W	188	0.02	0.94	K	268	0.07	0.96	W
68	0.06	0.84	F	209	0.05	0.94	L	282	0.07	0.96	R
72	0.08	0.93	N	220	0.03	0.94	E	286	0.06	0.96	R
77	0.06	0.93	V	223	0.05	0.94	L	308	0.09	0.84	A
78	0.01	0.93	P	229	0.06	0.82	E	309	0.06	0.82	F
79	0.06	0.93	V	238	0.04	0.97	W	310	0.04	0.82	R
93	0.02	0.93	I	239	0.04	0.97	L	331	0.09	0.89	G
96	0.05	0.94	Y	247	0.05	0.97	A				

One intuitive approach to search for functionally important sites is to focus on residues involved in ligand binding, *i*.*e*. residues that are located in the active centre. However, the question of whether GDAP1 is an active GST, or even lacks glutathione binding completely, has not been clearly resolved. Hexadecanedioic acid has been identified as possible ligand and subsequently co-crystallised with GDAP1 [11]. Gln235, Trp238, Arg282, Arg286, and Lys287 contact the ligand; Trp238, Arg282 and Arg286 are highly conserved (**Table 4**). This suggests that they could be important for ligand binding and that mutations in these positions could affect GDAP1 function; indeed, Trp238 and Arg282 are CMT mutation sites. These 3 residues cluster on the side of GDAP1, which must face the MOM surface. One option is that the bound fatty acid, in fact, mimics the membrane surface, and that these residues are directly involved in membrane surface binding at the outer surface of the MOM.

**Table 5** shows the entropy calculated for a combined data set (GDAP1, GDAP1L1, and GST). Since the GST superfamily is so diverse, we identified sites with entropy < 0.2 (less conserved than the calculation within a family). The 14 sites that are conserved and present in at least 70% of the sequences are listed in **Table 5**. These include Leu239 and Gly241 in the ⍺6-⍺7 loop and Ala247 and Asp 248 on helix ⍺7. The effect of the A247V mutation on protein stability may have been therefore expected based on its conservation across this very broad set of GSTs.

**Table 5:** Entropy analysis for the large data set including the GST dataset. Shown are residue positions with the lowest entropy (S < 0.2).

residue	entropy	frac	Amino acid
28	0.12	0.92	L
72	0.15	0.98	N
77	0.17	0.98	V
78	0.01	0.98	P
93	0.05	0.98	I
96	0.10	0.98	Y
109	0.17	0.9	L
111	0.14	0.81	P
223	0.10	0.89	L
239	0.20	0.9	L
241	0.14	0.9	G
247	0.12	0.9	A
248	0.02	0.9	D
286	0.17	0.86	R

### Kullback-Leibler divergence

**Table 6** shows the sites with a high KL divergence. These are those which are conserved in at least one group and different in the second group. The results have not been filtered by ignoring gaps since an insertion or deletion is of interest. Of the GDAP1 residues Cys51, Gly83, Tyr124, and Glu228 that are not assigned to a gap symbol, Cys51 and Gly83 are located close to the dimerisation site of GDAP1. Gly83 is a target of a non-pathological polymorphism, G83A [66]. The β sheet is the dimerisation site of GDAP1, and the larger amino acids Phe and Asp found in canonical GSTs may lead to steric hindrance during dimer formation. On the other hand, Phe and Asp are predestined for stacking and polar interactions respectively. Since the binding site for glutathione in GST takes place near this segment, these residues could be important for ligand binding in the GSTs, which may not be relevant for GDAP1, due to the apparent lack of glutathione binding. The observations support the assumption that the binding site for a small-molecule ligand, if any, in GDAP1 may be located elsewhere.

**Table 6:** KL divergence. KL_1_ and KL_2_ are the Kullback-Leibler divergence using the GDAP1 or GST group as reference distribution. S_1_ and S_2_ are the sequence entropy within the two groups. GDAP1 and GST show the most common residue within each group.

res num	KL_1_	KL_2_	*S*_1_	*S*_2_	GDAP1	GST
51	1.03	0.78	0.19	0.46	C	F
83	0.71	0.86	0.42	0.37	G	D
106	1.61	1.81	0.47	0.27	T	-
124	1.17	1.25	0.20	0.39	Y	W
131	0.97	2.91	0.11	0.16	L	-
132	0.94	1.81	0.19	0.40	P	-
149	0.73	2.12	0.27	0.26	D	-
150	0.70	1.80	0.22	0.28	S	-
151	1.84	2.23	0.34	0.37	M	-
166	1.28	2.32	0.26	0.26	N	-
186	1.13	2.43	0.42	0.29	I	-
192	1.05	1.88	0.25	0.47	L	-
193	0.87	2.32	0.46	0.47	K	-
228	1.16	1.99	0.41	0.39	E	Q
229	1.60	1.95	0.06	0.48	E	-
233	2.17	2.27	0.43	0.31	E	-
234	2.15	2.63	0.49	0.00	G	-
236	2.18	2.92	0.49	0.00	Q	-
295	1.55	2.37	0.45	0.49	H	-
300	2.44	2.51	0.13	0.41	L	-
319	1.05	3.00	0.44	0.22	V	-
339	1.54	1.30	0.48	0.35	L	-
352	2.52	1.62	0.09	0.42	R	-
353	1.32	1.76	0.40	0.47	P	-
355	2.27	1.90	0.36	0.00	P	-
356	2.30	2.00	0.32	0.41	N	-
358	2.50	2.58	0.14	0.00	F	-

On the other hand, Tyr124 and Glu228 in GDAP1 are close to each other in 3D space, being located on the core helices ⍺3 and ⍺6, respectively. The conversion of tyrosine to tryptophan (Tyr124 → Trp) and glutamic acid to glutamine (Glu228 → Gln) between GDAP1 and canonical GSTs causes only small changes in physicochemical properties. However, the central location of these residues in the GDAP1 fold suggests this finding may reflect a structural or functional aspect specific to GDAP1.

Of additional interest are Lys193 and Asn166, which are both close to the GDAP1-specific insertion. In contrast to the crystal structure, where Asn166 is not visible, the AlphaFold2 model predicts that the helix ⍺6 is bent, which brings Lys193 and Asn166 into close proximity. This could enable intramolecular interactions or provide a specific site for protein-protein interactions. Overall, the results indicate that certain subdomains in GDAP1 may have evolved to fulfill a different function compared to canonical GSTs.

## Discussion

Mutations in dozens of genes are causative for various subtypes of CMT disease. For most of them, the effects at the molecular level are not known, but one could even say that the working of the native protein is often poorly understood. One of the characterised examples is myelin protein P2, which loses thermal stability upon all the known 6 CMT mutations in the protein, while the crystal structure remains nearly unaltered [57, 58]. Furthermore, the disordered tail of myelin protein P0 is a target for P0 mutations, and its membrane interactions, inducing folding into helical structure, may be compromised upon CMT [67]. Periaxin carries several CMT-linked truncation mutations that abolish protein-protein interactions [68, 69], most notably with β4 integrin [70]. While these proteins, highly enriched in myelinating Schwann cells, are involved in the classical Schwann cell phenotypes related to myelination, it is evident that compromised mitochondrial function is one underlying cause of especially axonal CMT subtypes, and mutations in GDAP1 are linked to mitochondrial dysfunction. Linked to this mechanism, recent data show that GDAP1 may be involved in interactions with the actin and tubulin cytoskeletons [9].

Mutations in the GST-like domain of GDAP1 have a broad pathological spectrum. The molecular basis remains unknown despite cellular observations confirming the causality of impaired mitochondrial dynamics. Accurate structural information is required to support these findings. Below, we shall discuss some implications of our findings to understand GDAP1 function at the molecular level and the effects of missense mutations therein.

### CMT mutations at the GDAP1 protein level

CMT-linked missense mutations are relatively common in the *GDAP1* gene compared to other CMT target genes, especially when the size of the protein is taken into consideration [22, 71]. With careful examination of structural models and using them as inputs for further bioinformatics analyses, the mutations are observed to cause subtle changes in intramolecular residue interaction networks, in line with experimental data. A comprehensive understanding of the effects of single mutations requires observation of the local structure coupled to experimental data at the protein and cellular levels.

Of the residues highlighted by the entropy analysis either within the GDAP1 sequence set or the entire GST set, several are targets for missense mutations. Here, we shall briefly compare selected CMT mutations in GDAP1. The immediate environment of each residue is considered to shed light on local effects of each mutation.

Tyr29 is conserved at the symmetry axis of the GDAP1 dimer, forming a H bond between OH groups of Tyr29 from the two protomers. Y29S [72] would both remove this polar interaction and make the dimer interface much less hydrophobic.

Leu239 is at the tip of the ⍺6-⍺7 loop, inserted into the structural core, and its mutation to Phe has been reported in CMT [73]. The side chain is close to those of Cys240 and Ala247, and hence, a larger residue at this position could similarly affect protein stability as A247V. Similar effects could be foreseen for the C240Y mutation [74]. Pro111 is in the ⍺2-⍺3 loop, close in 3D space to the N terminus of helix ⍺7 and the ⍺6-⍺7 loop. Its mutation to His [75] would cause steric hindrance and alter the conformation of the ⍺2-⍺3 loop, possibly destabilising the fold.

Ala247, studied in the current work, lies centrally in the GDAP1 fold on helix ⍺7. A247V is linked to CMT, showing that no larger residue fits into this tight space and A247V destabilised the GDAP1 fold. Ala247 is one of the most conserved residues in the GST superfamily, indicating a role in the GST fold. Other residues conserved in the GDAP1 set include Leu255 and Gly262, which lie in the middle and at the end of helix ⍺7, respectively. L255F [76] would be expected to cause similar steric hindrance as A247V, and G262E [72] will likely disturb the tight turn right after helix ⍺7 and cause steric clashes. Both Leu255 and Gly262 are highly conserved in GDAP1 sequences.

Arg282, studied here in the form of the CMT mutation R282H, is one of the most conserved residues in the GDAP1/GDAP1L1 subfamily. Its strong hydrogen bonding to the carbonyl groups in the ⍺6-⍺7 loop indicates a central role in GDAP1 structure. In addition to R282H, the mutation R282C has been reported [77], and we can expect it to have the same kind of effect as R282H, losing the side-chain interactions of Arg282 to the backbone of the ⍺6-⍺7 loop. Arg282 is, in addition, optimally stacked against the aromatic side chain of Trp238, which is both a conserved residue and a CMT mutation target itself [78]. This Arg-Trp-(⍺6-⍺7) unit is likely important for the stable folding of GDAP1.

**Fig 8:**
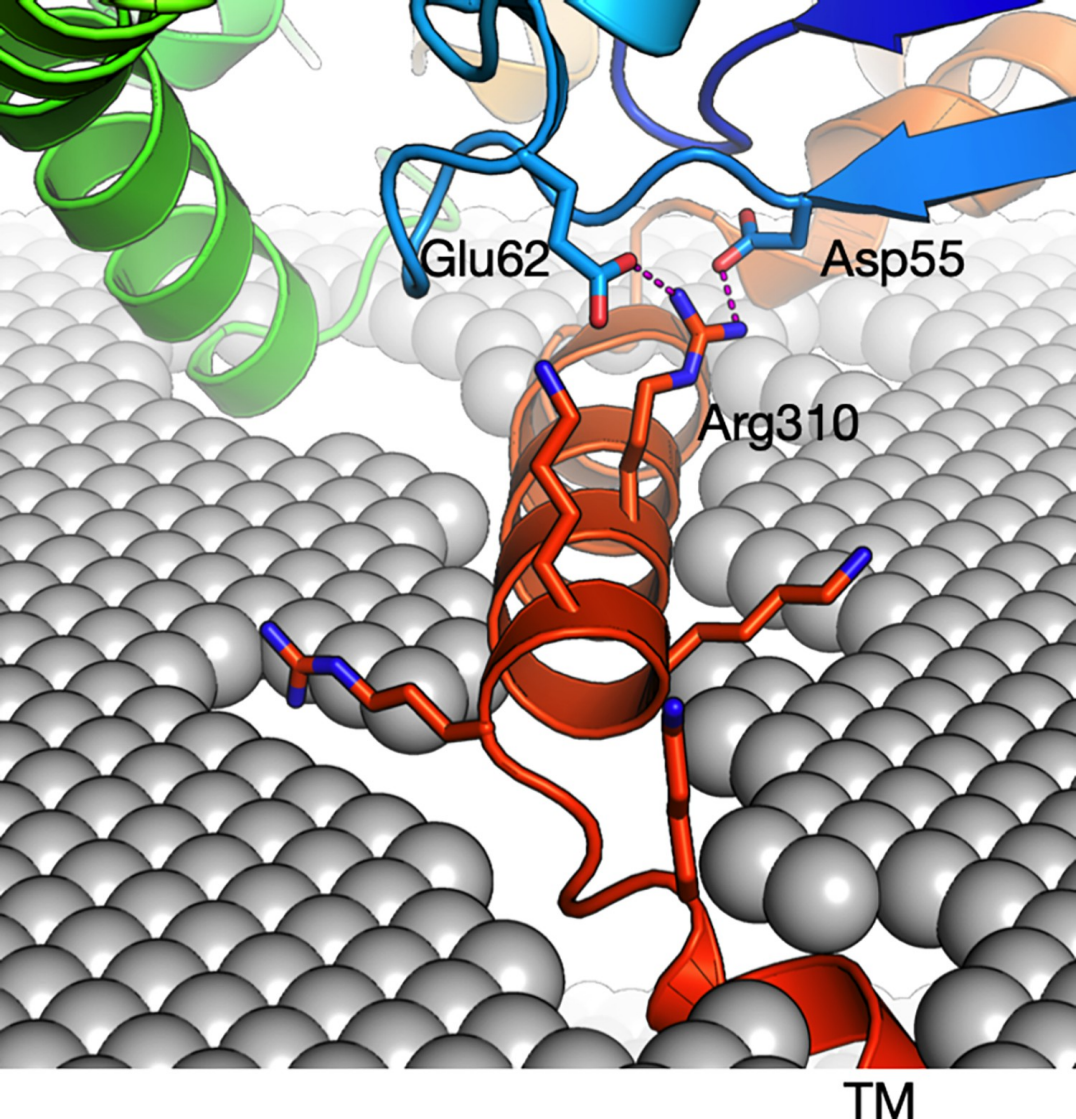
Location of Arg310 outside the GST-like core. Arg310 within the amphipathic helix preceding the transmembrane domain is predicted to make salt bridges back towards the core domain.

Arg310 is not present in the constructs we used for crystallisation, and hence, we have no high-resolution data on its conformation. However, the AlphaFold2 model of GDAP1 extends our crystal structures and predicts that Arg310 points away from the MOM surface and forms two salt bridges to acidic residues in the GDAP1 fold (**Fig 8**). Interestingly, Arg310 resides in the segment originally coined the hydrophobic domain; in our view, this fragment corresponds to an amphipathic helix, which could bind onto a phospholipid membrane surface. The AlphaFold2 prediction supports this view. Arg310 is likely to link this membrane-bound helix to the folded core of GDAP1 and, therefore, directly affect the conformation of full-length, membrane-bound GDAP1. This is supported by the lack of a destabilising effect on recombinant GDAP1 structure in our experiments; Arg310 is located outside the folded core and has a more subtle role.

The clinical profiling of CMT patients and experiments in cell-based models have shown that mutation of residues located within or near the vicinity of the transmembrane helix are severe [79, 80]. These mutations likely affect the proper localisation into the MOM, leading to impaired GDAP1 folding and function. So far, studies *in vitro* on the GDAP1 protein have been done only on soluble constructs, lacking the transmembrane domain. The stability of full-length GDAP1 *in vivo* will also involve interactions with the lipid bilayer, which is a topic of ongoing work.

### The central role of the ⍺6-⍺7 loop

Despite the broad spectrum of CMT disease mutations affecting GDAP1, certain general conclusions hold. The intramolecular interaction network described here shows that the critical helices in the C-terminal GST-like core domain are linked by residues that correspond to CMT target locations and/or are conserved in evolution. Therefore, these residues are likely essential for the structural integrity of GDAP1 and its function.

**Fig 9:**
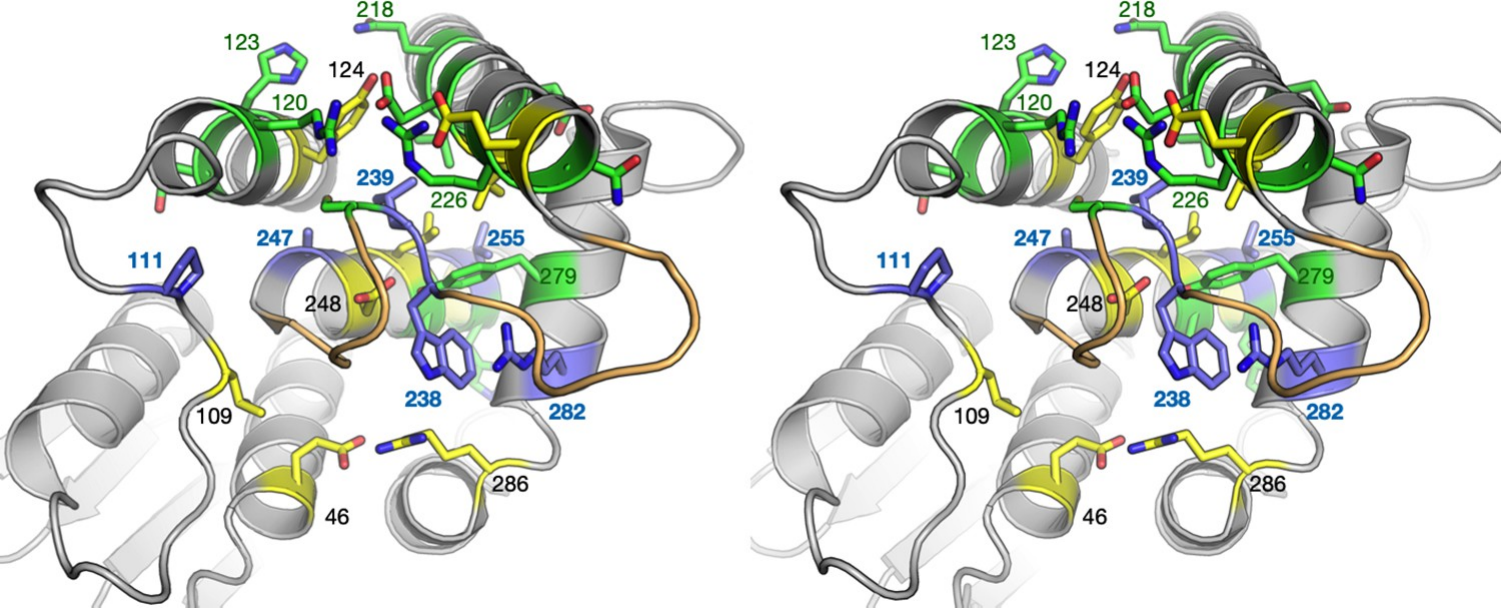
An overview of the ⍺6-⍺7 loop and its surroundings in light of known CMT mutations and conserved positions. In this stereo view, the ⍺6-⍺7 loop is coloured orange, green shows positions for CMT mutations, yellow positions that are highly conserved, and blue the positions that are both targeted by CMT mutations and highly conserved. Selected residues are labelled for clarity.

The ⍺6-⍺7 loop is a central feature in the structure of GDAP1, but also in all canonical GSTs. This loop inserts itself back into the protein core, being a central interaction hub between helices ⍺3, ⍺6, ⍺7, and ⍺8 (**Fig 9**). In GDAP1, the 3 residues at the tip of the loop (238–240) are all targets for CMT disease mutations, highlighting the crucial importance of this segment. Our entropy analysis confirms the strong conservation of this segment. We can then take a broader scope and further look at residues interacting with the ⍺6-⍺7 loop in GDAP1. Of note, several of the residues in direct contact with the ⍺6-⍺7 loop are known CMT mutation target sites and/or highly conserved (**Table 4**). Hence, the inward-bending ⍺6-⍺7 loop appears to be a central structural feature in GDAP1, and its alteration either directly or *via* disturbance of intramolecular interactions may be a common mechanism for CMT.

### GDAP1 as a member of the GST superfamily

The predictions that GDAP1 would be structurally related to GSTs have been confirmed by recent structural studies by us and others [10–12]. Important differences to canonical GSTs exist, however. The sequence identity between GDAP1 and any enzyme with known GST activity is very low, and contradictory results have been obtained as to the GST activity of GDAP1. In our hands, GDAP1 does not bind glutathione or act as a GST [11]. The latter is logical, since the GDAP1 dimers are formed completely differently from canonical GST, in which the active site in fact lies at the dimer interface [81]. We hypothesise that the residues conserved across the complete GDAP1/GST set are crucial for correct folding. On the other hand, residues additionally highlighted in the GDAP1/GDAP1L1 dataset may relate to GDAP1-specific functions and/or unique structural aspects of this subfamily.

Our conservation analyses highlight the low conservation of GDAP1 (and GDAP1L1) in the GST superfamily. While the entire evolutionary pathway cannot be traced based on the analyses, it is intriguing that GDAP1 is closer to bacterial than eukaryotic GSTs. It is possible that during evolution, GDAP1 has lost the characteristic GST activity, while becoming an integral membrane protein of the MOM. Its functions could, therefore, be mediated through protein-protein interactions instead of enzymatic activity. The functions can be redox-regulated, which could explain the observation that the human GDAP1 dimer is mediated by a disulphide bridge *via* Cys88 [11].

**Fig 10:**
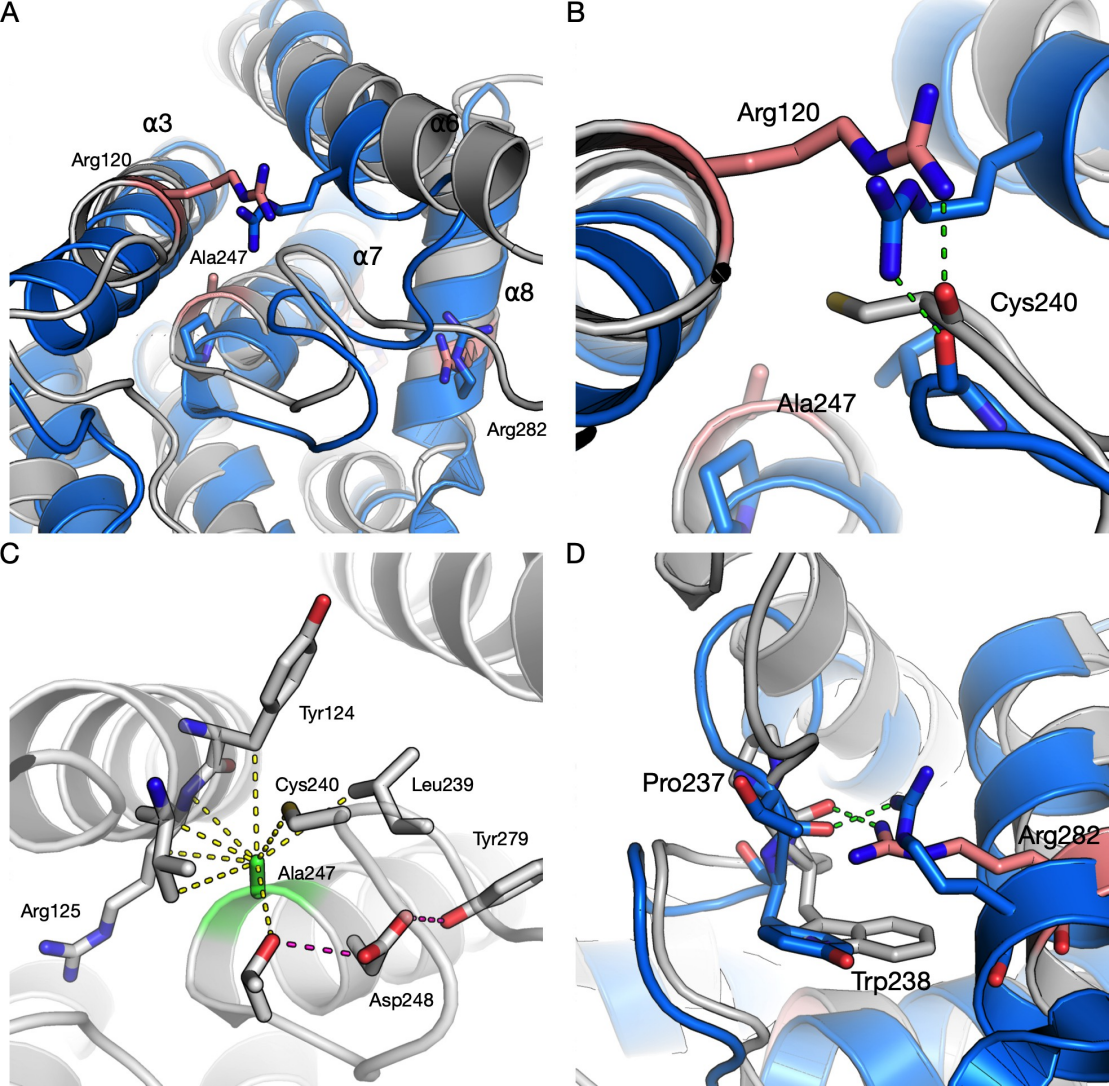
Comparisons of the studied mutations to a canonical GST from S. japonicum. GDAP1 is in gray and GST in blue. A. Overall view of the GDAP1-GST superposition, with the CMT mutation sites crystallised here highlighted in pink. B. Arg120 (pink) in GDAP1 and the corresponding interaction in GST, made by an Arg from a nearby helix. C. Tight packing of Ala247 (green) in the GDAP1 structure; Ala at this position is highly conserved across the whole GST family. Note Tyr124, which was highlighted in the KL divergence analysis, making direct van der Waals contact with Ala247. D. The interactions of Arg282 (pink) towards the ⍺6-⍺7 loop and Trp238 are conserved in GST.

To complement the above analyses, we superposed crystal structures of human GDAP1 and a canonical GST, that from *S*. *japonicum* [81], and analysed the current GDAP1 crystal structures with respect to GST. This is the GST widely used in molecular biology applications as a fusion tag for affinity purification. We were interested in the residues affected by CMT mutations, especially those crystallised here. Hence, of specific interest were the apparent non-conservation of Arg120 and the conservation of Ala247 and Arg282 (**Fig 10A**).

Arg120 in GDAP1 is a target of several CMT mutations [82–84], and its interactions with the backbone carbonyl of Cys240 in the ⍺6-⍺7 loop appear central. Structures of the mutants R120Q and R120W indicate loosening of the structure locally, as well as loss of the hydrogen bond, which is accompanied by a decrease in observed heat stability in R120Q and R120W [12]. Arg120 is not conserved in GSTs at the sequence level; however, an Arg residue from the neighbouring helix in GST reaches the same position and makes similar interactions to the same carbonyl group in the ⍺6-⍺7 loop (**Fig 10B**). These observations highlight the importance of the central ⍺6-⍺7 loop in intramolecular interaction networks and fold stability of GDAP1.

Ala247 of GDAP1, although perhaps a mundane residue *per se*, appears surprisingly conserved in the GST superfamily in our dataset (**Table 4**). In the superposed individual GST structure (**Fig 10A**), this residue is Pro, which fits well into the structure due to slightly different conformations of the main helices in GST. In GDAP1, Ala247 is so snugly packed (**Fig 10C**) that even the addition of two methyl groups in the A247V variant causes protein instability and disease, despite only minor effects in the crystal state. A247V is an example of a mutation introduced into the hydrophobic core that may have long-range effects on the entire protein structure stability. One of the residues in GDAP1 highlighted by the KL divergence analysis, Tyr124, closely interacts with Ala247, indicating a GDAP1-specific arrangement at this site. Additionally, one of the most conserved GDAP1 residues, Asp248, is central in a hydrogen bonding network linking the conserved CMT target Tyr279 into the picture (**Fig 10C**).

GST has an Arg corresponding to Arg282, and this residue is highly conserved. Arg282 in GDAP1 interacts directly with the backbone of the ⍺6-⍺7 loop, and a similar interaction is observed in GST (**Fig 10D**). This is another example of an Arg–loop backbone interaction in GDAP1 that is both conserved and relevant for human disease mutations.

### Biological implications

The function of GDAP1 at the molecular level remains enigmatic. While indications exist from functional studies [2, 3, 80], evidence is still incomplete for any enzymatic activity as well as direct protein-protein interactions. Redox regulation seems to play a role [9, 74, 85], but is this related to an enzymatic GST-like activity, or regulation of oligomeric state and/or protein-protein interactions?

At the molecular level, we believe to have identified important residue interaction networks between the core helices in the GST-like domain of GDAP1, strongly interacting with the inward ⍺6-⍺7 loop. These networks could be important for both GDAP1 stability and its interactions with other molecules, such as the membrane or the cytoskeleton. The correct conformation of GDAP1 on the MOM, as well as its interactions with other proteins, will then directly or indirectly affect mitochondrial dynamics to promote correct development and function of the nervous system. The disease mutations may–due to their involvement in the same intramolecular networks–cause similar overall effects on GDAP1 stability and properties, which then leads to the CMT disease phenotype in patients. In line with this, all the missense mutations we have studied at the protein level allow for GDAP1 folding, while at the same time decreasing the stability of the fold.

It should be noted that due to the large pool of GDAP1 mutants causing CMT, our experimental sample size is still relatively small, and the hypothesis may not be correct in all cases. However, we have picked mutations from different core secondary structure elements for experimental analyses to account for an incomplete dataset, and predictions have been done for all mutations [12], indicating a general trend of structural destabilisation upon CMT missense mutations in GDAP1.

### Concluding remarks

Considering mitochondrial dynamics and interactions with other organelles, the homology to GSTs brings attractive prospects for GDAP1 function. GDAP1 takes part not only in mitochondrial fission and fusion, but also in interactions with the endoplasmic reticulum, peroxisomes, Golgi, and the cytoskeleton. These findings coupled with structural and biophysical data shall aid the understanding of the pathophysiological mechanism of GDAP1-linked CMT and may affect future treatment approaches. Future studies are needed to identify proteins and small molecules directly interacting with full-length GDAP1 in the physiological setting, allowing further structural investigations on the related molecular processes in nervous system function and disease. In a wider setting, we hypothesise that decrease in overall protein stability upon missense mutations is one common mechanism for CMT at the molecular level.

## Supporting information

S1 Fig Batch mode SAXS analysis of wild-type GDAP1 and four CMT mutants. A. Scattering curves. B. SAXS parameters.
(PDF)

S2 Fig Uncropped, unedited gels used for the panels in Fig 4E. (PDF)

## References

[1] Watts ME, Pocock R, Claudianos C. Brain Energy and Oxygen Metabolism: Emerging Role in Normal Function and Disease. Front Mol Neurosci. 2018;11:216. Epub 20180622. doi: 10.3389/fnmol.2018.00216 ; PubMed Central PMCID: PMC6023993. 29988368 PMC6023993

[2] Barneo-Munoz M, Juarez P, Civera-Tregon A, Yndriago L, Pla-Martin D, Zenker J, et al. Lack of GDAP1 induces neuronal calcium and mitochondrial defects in a knockout mouse model of charcot-marie-tooth neuropathy. PLoS Genet. 2015;11(4):e1005115. Epub 20150410. doi: 10.1371/journal.pgen.1005115 ; PubMed Central PMCID: PMC4393229. 25860513 PMC4393229

[3] Miressi F, Benslimane N, Favreau F, Rassat M, Richard L, Bourthoumieu S, et al. GDAP1 Involvement in Mitochondrial Function and Oxidative Stress, Investigated in a Charcot-Marie-Tooth Model of hiPSCs-Derived Motor Neurons. Biomedicines. 2021;9(8). Epub 20210802. doi: 10.3390/biomedicines9080945 ; PubMed Central PMCID: PMC8393985. 34440148 PMC8393985

[4] Pla-Martin D, Rueda CB, Estela A, Sanchez-Piris M, Gonzalez-Sanchez P, Traba J, et al. Silencing of the Charcot-Marie-Tooth disease-associated gene GDAP1 induces abnormal mitochondrial distribution and affects Ca2+ homeostasis by reducing store-operated Ca2+ entry. Neurobiol Dis. 2013;55:140–51. Epub 20130328. doi: 10.1016/j.nbd.2013.03.010 . 23542510

[5] Rossi A, Pizzo P, Filadi R. Calcium, mitochondria and cell metabolism: A functional triangle in bioenergetics. Biochim Biophys Acta Mol Cell Res. 2019;1866(7):1068–78. Epub 20181026. doi: 10.1016/j.bbamcr.2018.10.016 . 30982525

[6] Niemann A, Ruegg M, La Padula V, Schenone A, Suter U. Ganglioside-induced differentiation associated protein 1 is a regulator of the mitochondrial network: new implications for Charcot-Marie-Tooth disease. J Cell Biol. 2005;170(7):1067–78. Epub 20050919. doi: 10.1083/jcb.200507087 ; PubMed Central PMCID: PMC2171517. 16172208 PMC2171517

[7] Niemann A, Wagner KM, Ruegg M, Suter U. GDAP1 mutations differ in their effects on mitochondrial dynamics and apoptosis depending on the mode of inheritance. Neurobiol Dis. 2009;36(3):509–20. Epub 20090925. doi: 10.1016/j.nbd.2009.09.011 . 19782751

[8] Cuesta A, Pedrola L, Sevilla T, Garcia-Planells J, Chumillas MJ, Mayordomo F, et al. The gene encoding ganglioside-induced differentiation-associated protein 1 is mutated in axonal Charcot-Marie-Tooth type 4A disease. Nat Genet. 2002;30(1):22–5. Epub 20011217. doi: 10.1038/ng798 . 11743580

[9] Wolf C, Pouya A, Bitar S, Pfeiffer A, Bueno D, Rojas-Charry L, et al. GDAP1 loss of function inhibits the mitochondrial pyruvate dehydrogenase complex by altering the actin cytoskeleton. Commun Biol. 2022;5(1):541. Epub 20220603. doi: 10.1038/s42003-022-03487-6 . 35662277 PMC9166793

[10] Googins MR, Woghiren-Afegbua AO, Calderon M, St. Croix CM, Kiselyov KI, VanDemark AP. Structural and functional divergence of GDAP1 from the glutathione S-transferase superfamily. The FASEB Journal. 2020;34:7192–207. doi: 10.1096/fj.202000110R 32274853 PMC9394736

[11] Nguyen GTT, Sutinen A, Raasakka A, Muruganandam G, Loris R, Kursula P. Structure of the Complete Dimeric Human GDAP1 Core Domain Provides Insights into Ligand Binding and Clustering of Disease Mutations. Frontiers in Molecular Biosciences. 2020;7:631232. doi: 10.3389/fmolb.2020.631232 33585569 PMC7873046

[12] Sutinen A, Nguyen GTT, Raasakka A, Muruganandam G, Loris R, Ylikallio E, et al. Structural insights into Charcot-Marie-Tooth disease-linked mutations in human GDAP1. FEBS Open Bio. 2022. Epub 20220504. doi: 10.1002/2211-5463.13422 . 35509130 PMC9249340

[13] Mannervik B, Alin P, Guthenberg C, Jensson H, Tahir MK, Warholm M, et al. Identification of three classes of cytosolic glutathione transferase common to several mammalian species: correlation between structural data and enzymatic properties. Proceedings of the National Academy of Sciences of the United States of America. 1985;82(21):7202–6. doi: 10.1073/pnas.82.21.7202 3864155 PMC390817

[14] Fontés M. Charcot Marie Tooth Disease. A Single Disorder? International Journal of Molecular Sciences. 2018;19(12). doi: 10.3390/ijms19123807 30501086 PMC6321061

[15] Rossor AM, Tomaselli PJ, Reilly MM. Recent advances in the genetic neuropathies. Current Opinion in Neurology. 2016;29(5):537–48. doi: 10.1097/WCO.0000000000000373 27584852 PMC5130159

[16] DiVincenzo C, Elzinga CD, Medeiros AC, Karbassi I, Jones JR, Evans MC, et al. The allelic spectrum of Charcot-Marie-Tooth disease in over 17,000 individuals with neuropathy. Mol Genet Genomic Med. 2014;2(6):522–9. Epub 20140821. doi: 10.1002/mgg3.106 ; PubMed Central PMCID: PMC4303222. 25614874 PMC4303222

[17] Szigeti K, Lupski JR. Charcot–Marie–Tooth disease. European Journal of Human Genetics. 2009;17(6):703–10. doi: 10.1038/ejhg.2009.31 19277060 PMC2947101

[18] Cassereau J, Chevrollier A, Gueguen N, Desquiret V, Verny C, Nicolas G, et al. Mitochondrial dysfunction and pathophysiology of Charcot-Marie-Tooth disease involving GDAP1 mutations. Experimental neurology. 2011;227(1):31–41. doi: 10.1016/j.expneurol.2010.09.006 20849849

[19] Mai P-T, Le D-T, Nguyen T-T, Le Gia H-L, Nguyen Le T-H, Le M, et al. Novel GDAP1 Mutation in a Vietnamese Family with Charcot-Marie-Tooth Disease. BioMed research international. 2019;2019:7132494. doi: 10.1155/2019/7132494 31179332 PMC6507255

[20] Zimon M, Baets J, Fabrizi GM, Jaakkola E, Kabzinska D, Pilch J, et al. Dominant GDAP1 mutations cause predominantly mild CMT phenotypes. Neurology. 2011;77(6):540–8. doi: 10.1212/WNL.0b013e318228fc70 21753178 PMC3272385

[21] Baxter RV, Ben Othmane K, Rochelle JM, Stajich JE, Hulette C, Dew-Knight S, et al. Ganglioside-induced differentiation-associated protein-1 is mutant in Charcot-Marie-Tooth disease type 4A/8q21. Nature genetics. 2002;30(1):21–2. doi: 10.1038/ng796 11743579

[22] Rzepnikowska W, Kochanski A. A role for the GDAP1 gene in the molecular pathogenesis of CharcotMarieTooth disease. Acta neurobiologiae experimentalis. 2018;78(1):1–13. 29694336

[23] Fagerberg L, Hallstrom BM, Oksvold P, Kampf C, Djureinovic D, Odeberg J, et al. Analysis of the human tissue-specific expression by genome-wide integration of transcriptomics and antibody-based proteomics. Molecular & cellular proteomics: MCP. 2014;13(2):397–406. doi: 10.1074/mcp.M113.035600 24309898 PMC3916642

[24] Dasari S, Gonuguntla S, Ganjayi MS, Bukke S, Sreenivasulu B, Meriga B. Genetic polymorphism of glutathione S-transferases: Relevance to neurological disorders. Pathophysiology. 2018;25(4):285–92. Epub 20180611. doi: 10.1016/j.pathophys.2018.06.001 . 29908890

[25] Kumar A, Dhull DK, Gupta V, Channana P, Singh A, Bhardwaj M, et al. Role of Glutathione-S-transferases in neurological problems. Expert Opin Ther Pat. 2017;27(3):299–309. Epub 20161110. doi: 10.1080/13543776.2017.1254192 . 27785931

[26] Pandey T, Chhetri G, Chinta R, Kumar B, Singh DB, Tripathi T, et al. Functional classification and biochemical characterization of a novel rho class glutathione S-transferase in Synechocystis PCC 6803. FEBS Open Bio. 2015;5:1–7. doi: 10.1016/j.fob.2014.11.006 25685659 PMC4309839

[27] Pflugmacher S, Wiegand C, Werner S, Schröder H, Kankaanpää H. Activity and substrate specificity of cytosolic and microsomal glutathione S-transferase in Australian black tiger prawns (Penaeus monodon) after exposure to cyanobacterial toxins. Environmental Toxicology. 2005;20(3):301–7. doi: 10.1002/tox.20121 15892065

[28] Studier FW. Protein production by auto-induction in high density shaking cultures. Protein expression and purification. 2005;41(1):207–34. doi: 10.1016/j.pep.2005.01.016 15915565

[29] Burkhardt A, Pakendorf T, Reime B, Meyer J, Fischer P, Stübe N, et al. Status of the crystallography beamlines at PETRA III. The European Physical Journal Plus. 2016;131(3):56. doi: 10.1140/epjp/i2016-16056-0

[30] Meents A, Reime B, Stuebe N, Fischer P, Warmer M, Goeries D, et al., editors. Development of an in-vacuum x-ray microscope with cryogenic sample cooling for beamline P11 at PETRA III. SPIE Optical Engineering + Applications; 2013 2013-9-26. San Diego, California, United States.

[31] Cianci M, Bourenkov G, Pompidor G, Karpics I, Kallio J, Bento I, et al. P13, the EMBL macromolecular crystallography beamline at the low-emittance PETRA III ring for high- and low-energy phasing with variable beam focusing. J Synchrotron Radiat. 2017;24(Pt 1):323–32. Epub 20170101. doi: 10.1107/S1600577516016465 ; PubMed Central PMCID: PMC5182027. 28009574 PMC5182027

[32] Kabsch W. XDS. Acta crystallographica Section D, Biological crystallography. 2010;66(Pt 2):125–32. doi: 10.1107/S0907444909047337 20124692 PMC2815665

[33] McCoy AJ, Grosse-Kunstleve RW, Adams PD, Winn MD, Storoni LC, Read RJ. ıt Phaser crystallographic software. Journal of Applied Crystallography. 2007;40(4):658–74. doi: 10.1107/S0021889807021206 19461840 PMC2483472

[34] Afonine PV, Grosse-Kunstleve RW, Echols N, Headd JJ, Moriarty NW, Mustyakimov M, et al. Towards automated crystallographic structure refinement with ıt phenix.refine. Acta Crystallographica Section D. 2012;68(4):352–67. doi: 10.1107/S0907444912001308 22505256 PMC3322595

[35] Emsley P, Lohkamp B, Scott WG, Cowtan K. Features and development of Coot. Acta crystallographica Section D, Biological crystallography. 2010;66(Pt 4):486–501. doi: 10.1107/S0907444910007493 20383002 PMC2852313

[36] Chen VB, Arendall WBr, Headd JJ, Keedy DA, Immormino RM, Kapral GJ, et al. MolProbity: all-atom structure validation for macromolecular crystallography. Acta crystallographica Section D, Biological crystallography. 2010;66(Pt 1):12–21. doi: 10.1107/S0907444909042073 20057044 PMC2803126

[37] Jumper J, Evans R, Pritzel A, Green T, Figurnov M, Ronneberger O, et al. Highly accurate protein structure prediction with AlphaFold. Nature. 2021;596(7873):583–9. Epub 20210715. doi: 10.1038/s41586-021-03819-2 ; PubMed Central PMCID: PMC8371605. 34265844 PMC8371605

[38] Huang J, Rauscher S, Nawrocki G, Ran T, Feig M, de Groot BL, et al. CHARMM36m: an improved force field for folded and intrinsically disordered proteins. Nat Methods. 2017;14(1):71–3. Epub 20161107. doi: 10.1038/nmeth.4067 ; PubMed Central PMCID: PMC5199616. 27819658 PMC5199616

[39] Jo S, Cheng X, Lee J, Kim S, Park SJ, Patel DS, et al. CHARMM-GUI 10 years for biomolecular modeling and simulation. J Comput Chem. 2017;38(15):1114–24. Epub 20161114. doi: 10.1002/jcc.24660 ; PubMed Central PMCID: PMC5403596. 27862047 PMC5403596

[40] Thureau A, Roblin P, Pérez J. BioSAXS on the SWING beamline at Synchrotron SOLEIL. J Appl Crystallogr. 2021;54:1698–710.

[41] Manalastas-Cantos K, Konarev PV, Hajizadeh NR, Kikhney AG, Petoukhov MV, Molodenskiy DS, et al. ATSAS 3.0: expanded functionality and new tools for small-angle scattering data analysis. J Appl Crystallogr. 2021;54(Pt 1):343–55. Epub 20210201. doi: 10.1107/S1600576720013412 ; PubMed Central PMCID: PMC7941305. 33833657 PMC7941305

[42] Konarev PV, Volkov VV, Sokolova AV, Koch MHJ, Svergun DI. PRIMUS: a Windows PC-based system for small-angle scattering data analysis. Journal of Applied Crystallography. 2003;36(5):1277–82. doi: 10.1107/S0021889803012779

[43] Svergun DI. Determination of the regularization parameter in indirect-transform methods using perceptual criteria. Journal of Applied Crystallography. 1992;25(4):495–503. doi: 10.1107/S0021889892001663

[44] Svergun DI, Petoukhov MV, Koch MHJ. Determination of domain structure of proteins from x-ray solution scattering. Biophys J. 2001;80:2946–53. doi: 10.1016/S0006-3495(01)76260-1 . 11371467 PMC1301478

[45] Svergun DI. Restoring low resolution structure of biological macromolecules from solution scattering using simulated annealing. Biophysical journal. 1999;76(6):2879–86. doi: 10.1016/S0006-3495(99)77443-6 10354416 PMC1300260

[46] Svergun DI, Barberato C, Koch MH. CRYSOL—A program to evaluate X-ray solution scattering of biological macromolecules from atomic coordinates. J Appl Crystallogr. 1995;28:768–73. doi: 10.1107/S0021889895007047

[47] Blanchet CE, Spilotros A, Schwemmer F, Graewert MA, Kikhney A, Jeffries CM, et al. Versatile sample environments and automation for biological solution X-ray scattering experiments at the P12 beamline (PETRA III, DESY). J Appl Crystallogr. 2015;48(Pt 2):431–43. Epub 20150312. doi: 10.1107/S160057671500254X ; PubMed Central PMCID: PMC4379436. 25844078 PMC4379436

[48] Miles AJ, Wallace BA. CDtoolX, a downloadable software package for processing and analyses of circular dichroism spectroscopic data. Protein science: a publication of the Protein Society. 2018;27(9):1717–22. doi: 10.1002/pro.3474 30168221 PMC6194270

[49] Altschul SF, Madden TL, Schaffer AA, Zhang J, Zhang Z, Miller W, et al. Gapped BLAST and PSI-BLAST: a new generation of protein database search programs. Nucleic Acids Res. 1997;25(17):3389–402. doi: 10.1093/nar/25.17.3389 ; PubMed Central PMCID: PMC146917. 9254694 PMC146917

[50] Katoh K, Misawa K, Kuma K, Miyata T. MAFFT: a novel method for rapid multiple sequence alignment based on fast Fourier transform. Nucleic Acids Res. 2002;30(14):3059–66. doi: 10.1093/nar/gkf436 ; PubMed Central PMCID: PMC135756. 12136088 PMC135756

[51] Kullback S, Leibler RA. On information and sufficiency. Ann Math Stat. 1951;22:79–86.

[52] Mackay DJC. Information theory, inference, and learning algorithms: Cambridge University Press; 2003.

[53] Huelsenbeck JP, Ronquist F. MRBAYES: Bayesian inference of phylogenetic trees. Bioinformatics. 2001;17:754–5 doi: 10.1093/bioinformatics/17.8.754 11524383

[54] Ronquist F, Teslenko M, van der Mark P, Ayres DL, Darling A, Hohna S, et al. MrBayes 3.2: efficient Bayesian phylogenetic inference and model choice across a large model space. Syst Biol. 2012;61(3):539–42. Epub 20120222. doi: 10.1093/sysbio/sys029 ; PubMed Central PMCID: PMC3329765. 22357727 PMC3329765

[55] Letunic I, Bork P. Interactive Tree Of Life (iTOL) v5: an online tool for phylogenetic tree display and annotation. Nucleic Acids Res. 2021;49(W1):W293–W6. doi: 10.1093/nar/gkab301 ; PubMed Central PMCID: PMC8265157. 33885785 PMC8265157

[56] Torda AE. sequtils 1.0. Zenodo. 2020:4066305. doi: 10.5281/zenodo.4066305

[57] Ruskamo S, Nieminen T, Kristiansen CK, Vatne GH, Baumann A, Hallin EI, et al. Molecular mechanisms of Charcot-Marie-Tooth neuropathy linked to mutations in human myelin protein P2. Sci Rep. 2017;7(1):6510. Epub 20170726. doi: 10.1038/s41598-017-06781-0 ; PubMed Central PMCID: PMC5529448. 28747762 PMC5529448

[58] Uusitalo M, Klenow MB, Laulumaa S, Blakeley MP, Simonsen AC, Ruskamo S, et al. Human myelin protein P2: from crystallography to time-lapse membrane imaging and neuropathy-associated variants. FEBS J. 2021;288(23):6716–35. Epub 20210714. doi: 10.1111/febs.16079 . 34138518

[59] Chung KW, Kim SM, Sunwoo IN, Cho SY, Hwang SJ, Kim J, et al. A novel GDAP1 Q218E mutation in autosomal dominant Charcot-Marie-Tooth disease. J Hum Genet. 2008;53(4):360–4. Epub 20080131. doi: 10.1007/s10038-008-0249-3 . 18231710

[60] Crimella C, Tonelli A, Airoldi G, Baschirotto C, D’Angelo MG, Bonato S, et al. The GST domain of GDAP1 is a frequent target of mutations in the dominant form of axonal Charcot Marie Tooth type 2K. J Med Genet. 2010;47(10):712–6. Epub 20100803. doi: 10.1136/jmg.2010.077909 . 20685671

[61] Kabzinska D, Kotruchow K, Cegielska J, Hausmanowa-Petrusewicz I, Kochanski A. A severe recessive and a mild dominant form of Charcot-Marie-Tooth disease associated with a newly identified Glu222Lys GDAP1 gene mutation. Acta Biochim Pol. 2014;61(4):739–44. Epub 20141022. . 25337607

[62] Moroni I, Morbin M, Milani M, Ciano C, Bugiani M, Pagliano E, et al. Novel mutations in the GDAP1 gene in patients affected with early-onset axonal Charcot-Marie-Tooth type 4A. Neuromuscul Disord. 2009;19(7):476–80. Epub 20090604. doi: 10.1016/j.nmd.2009.04.014 . 19500985

[63] Laimer J, Hiebl-Flach J, Lengauer D, Lackner P. MAESTROweb: a web server for structure-based protein stability prediction. Bioinformatics. 2016;32(9):1414–6. doi: 10.1093/bioinformatics/btv769 WOS:000376106100021. 26743508

[64] Parthiban V, Gromiha MM, Schomburg D. CUPSAT: prediction of protein stability upon point mutations. Nucleic Acids Res. 2006;34(Web Server issue):W239–42. doi: 10.1093/nar/gkl190 ; PubMed Central PMCID: PMC1538884. 16845001 PMC1538884

[65] Spang A, Saw JH, Jorgensen SL, Zaremba-Niedzwiedzka K, Martijn J, Lind AE, et al. Complex archaea that bridge the gap between prokaryotes and eukaryotes. Nature. 2015;521(7551):173–9. Epub 20150506. doi: 10.1038/nature14447 ; PubMed Central PMCID: PMC4444528. 25945739 PMC4444528

[66] Pedrola L, Espert A, Valdes-Sanchez T, Sanchez-Piris M, Sirkowski EE, Scherer SS, et al. Cell expression of GDAP1 in the nervous system and pathogenesis of Charcot-Marie-Tooth type 4A disease. J Cell Mol Med. 2008;12(2):679–89. Epub 20071116. doi: 10.1111/j.1582-4934.2007.00158.x ; PubMed Central PMCID: PMC2570022. 18021315 PMC2570022

[67] Raasakka A, Ruskamo S, Barker R, Krokengen OC, Vatne GH, Kristiansen CK, et al. Neuropathy-related mutations alter the membrane binding properties of the human myelin protein P0 cytoplasmic tail. PLoS One. 2019;14(6):e0216833. Epub 20190607. doi: 10.1371/journal.pone.0216833 ; PubMed Central PMCID: PMC6555526. 31173589 PMC6555526

[68] Sherman DL, Brophy PJ. A murine model of Charcot-Marie-Tooth disease 4F reveals a role for the C-terminus of periaxin in the formation and stabilization of Cajal bands. Wellcome Open Res. 2018;3:20. Epub 20180301. doi: 10.12688/wellcomeopenres.13673.1 ; PubMed Central PMCID: PMC5861512. 29623298 PMC5861512

[69] Marchesi C, Milani M, Morbin M, Cesani M, Lauria G, Scaioli V, et al. Four novel cases of periaxin-related neuropathy and review of the literature. Neurology. 2010;75(20):1830–8. doi: 10.1212/WNL.0b013e3181fd6314 . 21079185

[70] Raasakka A, Linxweiler H, Brophy PJ, Sherman DL, Kursula P. Direct Binding of the Flexible C-Terminal Segment of Periaxin to beta4 Integrin Suggests a Molecular Basis for CMT4F. Front Mol Neurosci. 2019;12:84. Epub 20190409. doi: 10.3389/fnmol.2019.00084 ; PubMed Central PMCID: PMC6465933. 31024253 PMC6465933

[71] Azzedine H, Ruberg M, Ente D, Gilardeau C, Perie S, Wechsler B, et al. Variability of disease progression in a family with autosomal recessive CMT associated with a S194X and new R310Q mutation in the GDAP1 gene. Neuromuscul Disord. 2003;13(4):341–6. . 12868504

[72] Nykamp K, Anderson M, Powers M, Garcia J, Herrera B, Ho YY, et al. Sherloc: a comprehensive refinement of the ACMG-AMP variant classification criteria. Genet Med. 2017;19(10):1105–17. Epub 20170511. doi: 10.1038/gim.2017.37 ; PubMed Central PMCID: PMC5632818. 28492532 PMC5632818

[73] Ammar N, Nelis E, Merlini L, Barisic N, Amouri R, Ceuterick C, et al. Identification of novel GDAP1 mutations causing autosomal recessive Charcot-Marie-Tooth disease. Neuromuscul Disord. 2003;13(9):720–8. doi: 10.1016/s0960-8966(03)00093-2 . 14561495

[74] Cassereau J, Chevrollier A, Codron P, Goizet C, Gueguen N, Verny C, et al. Oxidative stress contributes differentially to the pathophysiology of Charcot-Marie-Tooth disease type 2K. Experimental neurology. 2020;323:113069. doi: 10.1016/j.expneurol.2019.113069 31655048

[75] Chung KW, Hyun YS, Lee HJ, Jung HK, Koo H, Yoo JH, et al. Two recessive intermediate Charcot-Marie-Tooth patients with GDAP1 mutations. J Peripher Nerv Syst. 2011;16(2):143–6. doi: 10.1111/j.1529-8027.2011.00329.x . 21692914

[76] Volodarsky M, Kerkhof J, Stuart A, Levy M, Brady LI, Tarnopolsky M, et al. Comprehensive genetic sequence and copy number analysis for Charcot-Marie-Tooth disease in a Canadian cohort of 2517 patients. J Med Genet. 2021;58(4):284–8. Epub 20200506. doi: 10.1136/jmedgenet-2019-106641 . 32376792

[77] Nelis E, Erdem S, Van Den Bergh PY, Belpaire-Dethiou MC, Ceuterick C, Van Gerwen V, et al. Mutations in GDAP1: autosomal recessive CMT with demyelination and axonopathy. Neurology. 2002;59(12):1865–72. doi: 10.1212/01.wnl.0000036272.36047.54 . 12499475

[78] Antoniadi T, Buxton C, Dennis G, Forrester N, Smith D, Lunt P, et al. Application of targeted multi-gene panel testing for the diagnosis of inherited peripheral neuropathy provides a high diagnostic yield with unexpected phenotype-genotype variability. BMC Med Genet. 2015;16:84. Epub 20150921. doi: 10.1186/s12881-015-0224-8 ; PubMed Central PMCID: PMC4578331. 26392352 PMC4578331

[79] Kabzinska D, Niemann A, Drac H, Huber N, Potulska-Chromik A, Hausmanowa-Petrusewicz I, et al. A new missense GDAP1 mutation disturbing targeting to the mitochondrial membrane causes a severe form of AR-CMT2C disease. Neurogenetics. 2011;12(2):145–53. Epub 20110302. doi: 10.1007/s10048-011-0276-7 . 21365284

[80] Rzepnikowska W, Kaminska J, Kabzinska D, Kochanski A. Pathogenic Effect of GDAP1 Gene Mutations in a Yeast Model. Genes (Basel). 2020;11(3). Epub 20200314. doi: 10.3390/genes11030310 ; PubMed Central PMCID: PMC7140815. 32183277 PMC7140815

[81] Kursula I, Heape AM, Kursula P. Crystal structure of non-fused glutathione S-transferase from Schistosoma japonicum in complex with glutathione. Protein Pept Lett. 2005;12(7):709–12. doi: 10.2174/0929866054696154 . 16522189

[82] Boerkoel CF, Takashima H, Nakagawa M, Izumo S, Armstrong D, Butler I, et al. CMT4A: identification of a Hispanic GDAP1 founder mutation. Ann Neurol. 2003;53(3):400–5. doi: 10.1002/ana.10505 . 12601710

[83] Claramunt R, Pedrola L, Sevilla T, Lopez de Munain A, Berciano J, Cuesta A, et al. Genetics of Charcot-Marie-Tooth disease type 4A: mutations, inheritance, phenotypic variability, and founder effect. Journal of medical genetics. 2005;42(4):358–65. doi: 10.1136/jmg.2004.022178 15805163 PMC1736030

[84] Manganelli F, Pisciotta C, Nolano M, Capponi S, Geroldi A, Topa A, et al. A novel autosomal dominant GDAP1 mutation in an Italian CMT2 family. J Peripher Nerv Syst. 2012;17(3):351–5. doi: 10.1111/j.1529-8027.2012.00414.x . 22971097

[85] Cantarero L, Juárez-Escoto E, Civera-Tregón A, Rodríguez-Sanz M, Roldán M, Benítez R, et al. Mitochondria-lysosome membrane contacts are defective in GDAP1-related Charcot-Marie-Tooth disease. Human Molecular Genetics. 2021;29(22):3589–605. doi: 10.1093/hmg/ddaa243 33372681 PMC7823109

