# Supplementary figures and images for: Conserved intramolecular networks in GDAP1 are closely connected to CMT-linked mutations and protein stability
Source: PLoS One. 2023 Apr 14;18(4):e0284532. doi: 10.1371/journal.pone.0284532 (PMC10104300; doi:10.1371/journal.pone.0284532)

A

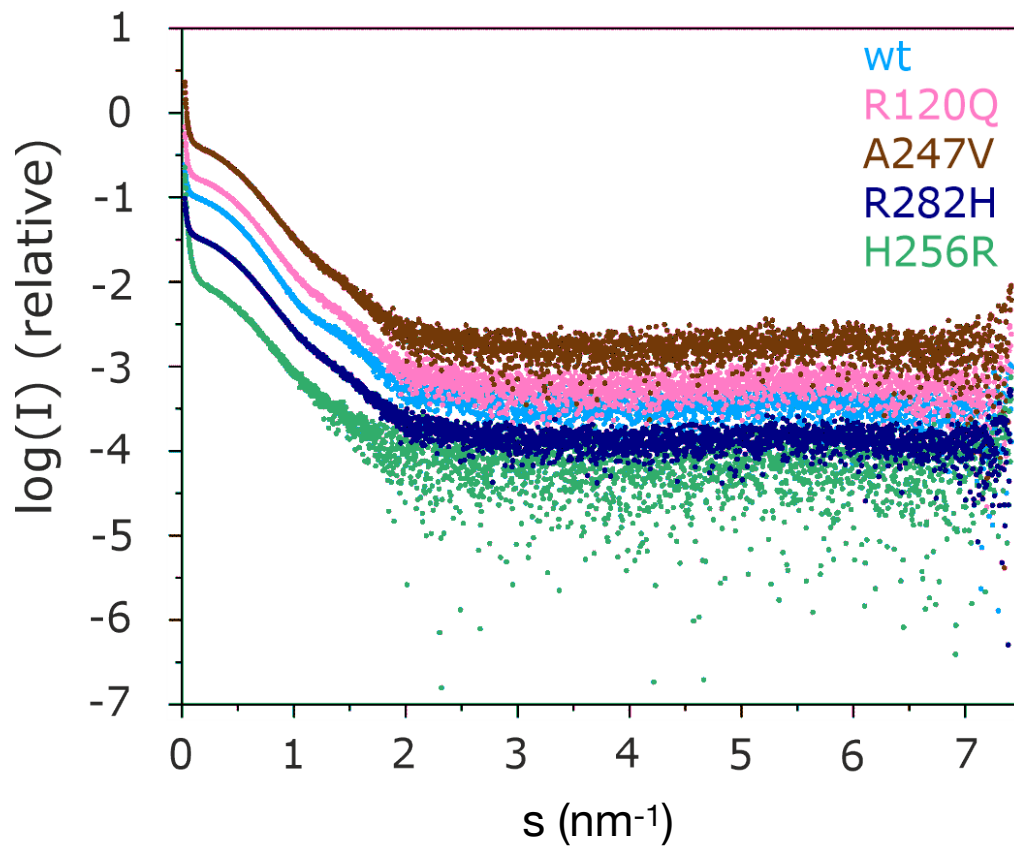

B

| sample          | $R_g$ (nm) | Porod volume ( $\text{\AA}^3$ ) | $D_{\text{max}}$ (nm) | C (mg/ml) |
|-----------------|------------|---------------------------------|-----------------------|-----------|
| Wild-type GDAP1 | 3.07       | 105552                          | 9.94                  | 4         |
| R120Q           | 3.02       | 95664                           | 9.81                  | 4         |
| A247V           | 3.02       | 91945                           | 9.80                  | 4         |
| H256R           | 3.02       | 94079                           | 8.68                  | 1.15      |
| R282H           | 3.01       | 93243                           | 9.54                  | 3         |

Supplement: S1 Fig — A. Scattering curves. B. SAXS parameters. (PDF) [file pone.0284532.s001.pdf]

A

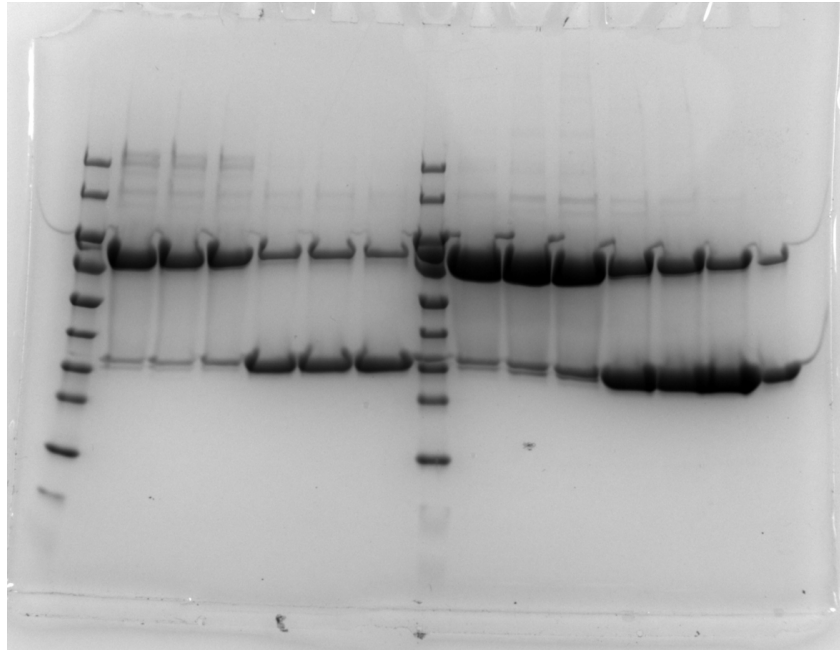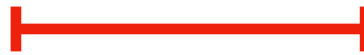

Fig4E - top panel

B

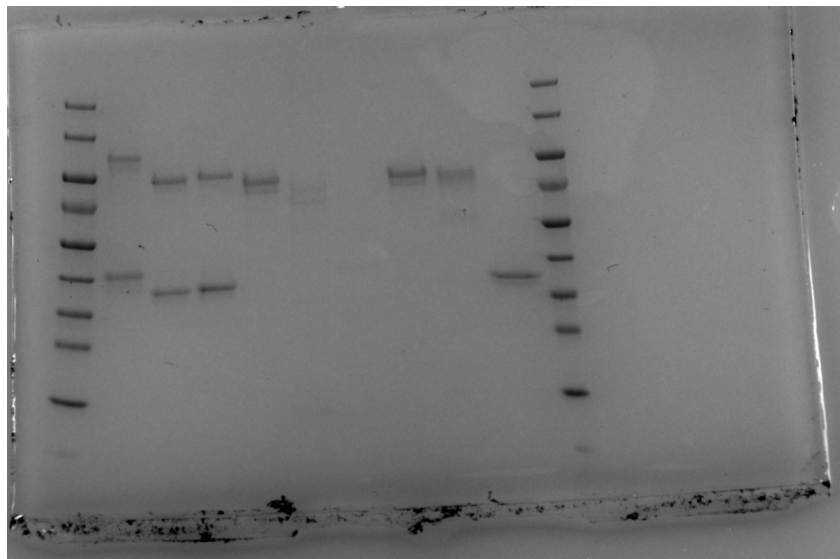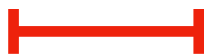

Fig4E - bottom panel

Supplement: S2 Fig — (PDF) [file pone.0284532.s002.pdf]
